# Targeting Respiratory Viruses: The Efficacy of Intranasal mRNA Vaccination in Generating Protective Mucosal and Systemic Immunity Against Influenza A (H1N1)

**DOI:** 10.1111/irv.70093

**Published:** 2025-03-24

**Authors:** Sara Yahyaei, Asghar Abdoli, Abbas Jamali, Ali Teimoori, Ehsan Arefian, Zohre Eftekhari, Parisa Jamur

**Affiliations:** ^1^ Hepatitis and AIDS Department Pasteur Institute of Iran Tehran Iran; ^2^ Student Research Committee Pasteur Institute of Iran Tehran Iran; ^3^ Department of Influenza and Other Respiratory Viruses Pasteur Institute of Iran Tehran Iran; ^4^ Department of Virology, Faculty of Medicine Hamadan University of Medical Sciences Hamadan Iran; ^5^ Department of Microbiology, School of Biology, College of Science University of Tehran Tehran Iran; ^6^ Biotechnology Department Pasteur Institute of Iran Tehran Iran

**Keywords:** intranasal, mannosylated chitosan, mRNA vaccine

## Abstract

Four significant influenza outbreaks have occurred over the past 100 years, and the 1918 influenza pandemic is the most severe. Since influenza viruses undergo antigenic evolution, they are the pathogens most likely to trigger a new pandemic shortly. Intranasal vaccination offers a promising strategy for preventing diseases triggered by respiratory viruses by eliciting an immunoglobulin A (IgA) response, limiting virus replication and transmission from the respiratory tract more efficiently than intramuscular vaccines. Combining intranasal administration and mRNA‐lipid nanoparticles can be an ideal strategy for limiting the extent of the next flu pandemic. This study explored the immunogenicity of intranasally delivered mRNA encapsulated in mannose‐histidine‐conjugated chitosan lipid nanoparticles (MHCS‐LNPs) as a vaccine against influenza A (H1N1) in BALB/c mice. Intranasal administration of mRNA‐MHCS‐LNPs resulted in the generation of influenza A (H1N1) hemagglutinin‐specific neutralizing antibodies in vaccinated animals. The enzyme‐linked immunosorbent assay (ELISA) results indicated a notable increase in the quantity of immunoglobulin G (IgG) and IgA antibodies in serum and the bronchoalveolar lavage fluid (BALF), respectively, and exhibited influenza A‐specific IFN‐γ secretion in vaccinated mice, as well as a noticeable alteration in IL‐5 production. Overall, this study demonstrated an effective immunogenic response against respiratory viral infections through intranasal delivery of an mRNA‐MHCS‐LNP vaccine.

## 1 Introduction

Based on World Health Organization's annual reports, seasonal influenza, caused by influenza A and B viruses, results in millions of severe illnesses and hundreds of thousands of deaths annually [1]. Pandemics are driven by influenza A due to its ability to infect both humans and animals [2] and its segmented RNA genome, which allows for antigenic shift and the emergence of new strains that evade existing immunity [3]. This constant evolution of the hemagglutinin (HA) protein makes influenza a significant and costly global health burden [4].

Compared to egg‐dependent vaccine platforms, mRNA vaccines offer advantages such as egg‐free and cell‐free production, elimination of egg‐adaptive mutations and immune responses against egg proteins or DNA vectors, no risk of integration into the host genome, and fast, scalable manufacturing process [5]. Regarding efficacy, clinical trials of approved mRNA vaccines have shown noticeable results through the induction of neutralizing antibodies and the activation of T cells [6, 7, 8, 9]. Studies have confirmed the rapid development of high levels of IgG and IgM antibodies with neutralizing abilities detectable for up to 6‐months postvaccination, as well as the presence of antigen‐specific CD4 and CD8 T cells up to 6 months after the initial vaccination [7, 10, 11]. Another key advantage of mRNA vaccine technology is based on the transient nature and short pharmacokinetic half‐life of RNA molecules, which prevents T‐cell depletion due to constant antigen exposure [12].

During in vitro transcription, modified nucleotides like N1‐methyl‐pseudouridine (m1Ψ) are incorporated into RNA. Replacing uridine with m1Ψ reduces TLR3 activation and inflammatory signaling [13]. This modification also alters translation by promoting ribosome pausing and increasing mRNA density [14].

Despite these advantages, in vivo administration of mRNA in the blood or tissues is restricted due to its structural instability. Encapsulation of mRNA within nanoparticles ensures efficient and safe delivery to the desired site and extends the transcript half‐life [15]. Cationic polymers, like chitosan, are widely used for nucleic acid delivery. They neutralize the negative charge of nucleic acids, improving cell membrane interaction and transfection efficiency [16]. Chitosan, a biodegradable and biocompatible polymer with low toxicity, enhances cell permeability, adheres to mucus, and degrades into nontoxic components, making it beneficial for gene delivery [17]. However, its low buffering capacity, insolubility at physiological pH, and meager rate of endosomal escape limit the application of chitosan as a gene carrier. Functionalizing chitosan with histidine, an amino acid containing an imidazole ring, increases endosomal escape through the proton‐sponge effect, which is an osmosis‐driven process and can cause the endosome to swell and eventually rupture, releasing the contents into the cytoplasm [18, 19, 20, 21].

Targeting antigen‐presenting cells (APCs) with surface‐modified nanoparticles enhances vaccine potential. Mannose‐modified chitosan nanoparticles exploit dendritic cell (DC) mannose receptors and boost antigen uptake and immune responses, including cytotoxic T lymphocyte (CTL) activation [22, 23, 24, 25].

Lipid nanoparticles (LNPs), particularly those with cationic phospholipids like DOTAP (1,2 Dioleyl‐3‐trimethylammonium propane), are widely used for gene delivery. They enhance cell binding and membrane fusion through electrostatic interactions with nucleic acids [26]. The beneficial characteristics of LNPs and chitosan, combined with their advantageous features, can develop ideal delivery nanoparticles.

At present, almost all approved and licensed vaccines against respiratory pathogens are administered intramuscularly and mainly induce systemic immune responses. However, since respiratory pathogens such as influenza and SARS‐CoV‐2 viruses can be transmitted from vaccinated individuals to others through infectious aerosols and droplets, local mucosal immune responses at virus entry sites, in addition to systemic immune responses, can minimize viral spread and limit infection [27]. Intranasal vaccination offers advantages like noninvasiveness, ease of use, and rapid immune response, making it ideal for mass vaccination. However, rapid mucociliary clearance hinders nanoparticle uptake and vaccine efficacy [28]. Given that chitosan nanoparticles are highly positively charged, interacting with nasal epithelial surfaces presents a potent mucoadhesive delivery system and increases antigen uptake. Following this notion, we developed mRNA‐chitosan LNPs encoding the full‐length influenza A (H1N1) prefusion‐stabilized hemagglutinin protein delivered by mannose‐histidine‐conjugated chitosan lipid nanoparticles (MHCS‐LNPs). Here, we present in vitro and in vivo evaluation data on the efficacy, safety, physicochemical properties, stability, and immunogenicity of mRNA‐MHCS‐LNPs in mice.

## 2 Materials and Methods

### 2.1 mRNA Vaccine Preparation

The full‐length gene and protein sequence for the HA protein of the influenza A (H1N1) virus were sourced from specific accession numbers (FJ966082 and ACP41105). The HA was engineered with proline substitutions and optimized for human codon use, placed under the T7 promoter in a plasmid. The construct was inserted into the PUC57 plasmid and transformed into *Escherichia coli* DH5α for amplification. A GFP gene was also included for reporter purposes. mRNA was transcribed using modified nucleotides and capped. For nanoparticle preparation, histidine was conjugated with chitosan to form HCS using EDC and NHS chemistry [29]. This was followed by mannose conjugation to HCS via reductive amination, resulting in MHCS [20, 30]. MHCS was used to form polyplexes with mRNA at various nitrogen‐to‐phosphate (N:P) ratios. These were then encapsulated into LNPs using a reverse‐phase evaporation method, involving lipids like cholesterol and phospholipid [31, 32]. The size, polydispersity, and zeta potential of the nanoparticles were measured using dynamic light scattering [33, 34]. Encapsulation efficiency was determined via fluorescence spectroscopy with SYBR Green II [35, 36]. The effectiveness of the mRNA‐MHCS‐LNPs was tested in cell lines (HEK 293T, A549, and RAW264.7) by assessing GFP expression through fluorescence microscopy and flow cytometry. Stability tests included monitoring encapsulation efficiency, size, and zeta potential over time when stored at different temperatures [37]. Cytotoxicity was evaluated using an XTT assay on HEK‐293T, RAW264.7, and A549 cells [38] (for details, see Supporting Information).

### 2.2 Animal Studies

Female BALB/c mice (6–8‐weeks old) were ethically sourced and housed under standard lab conditions. Sixty‐four mice were divided into six groups of eight to evaluate mRNA‐MHCS‐LNPs: three intranasal groups (6‐, 12‐, and 24‐μg mRNA), one intramuscular group (6‐μg mRNA), a PBS control, and a commercial influenza vaccine control. Intranasal administration was performed under anesthesia. A booster dose was given on Day 14. At Week 4, blood and BALF samples were collected, and spleens were harvested for analysis. The study was approved (IR.PII.REC.1400.067 on December 11, 2021) and followed ARRIVE guidelines [39, 40] (for details, see Supporting Information).

### 2.3 Evaluation of Humoral, Cellular, and Mucosal Immune Responses

Influenza A (H1N1) hemagglutinin‐specific IgG titers in serum were measured by indirect enzyme‐linked immunosorbent assay (ELISA) [41]. Splenocyte IL‐5 and IFN‐γ concentrations, indicative of cell‐mediated immunity, were assessed by ELISA after stimulating splenocytes with hemagglutinin peptides [42]. Mucosal IgA responses in BALF were also measured using an H1N1 hemagglutinin‐specific IgA ELISA. All ELISAs used absorbance readings at 450 nm (with a 630‐nm reference for IgA) to determine antibody/cytokine concentrations [43, 44] (for details, see Supporting Information).

### 2.4 Hemagglutination Inhibition Assay

Hemagglutination inhibition (HI) assays were performed using standard procedures. Chicken erythrocytes were washed and prepared as a 0.5% suspension. Formaldehyde‐inactivated influenza strains were serially diluted in a 96‐well plate to determine the hemagglutination assay (HA) titration endpoint, and is conducted to measure the amount of virus particles required for hemagglutination and is reported in HA units. For the HI assay, mice sera were diluted, incubated with virus containing four HA units, and then mixed with chicken RBCs. The HI titer, defined as the final serum dilution at which complete HI was observed, was visually determined [45, 46, 47, 48] (for details, see Supporting Information).

### 2.5 Statistical Analysis

The collected information was analyzed using GraphPad Prism 10 software. The t‐test and one‐way ANOVA were used to analyze the results statistically, as needed. A *p*‐value lower than 0.05 was considered to indicate a significant difference.

## 3 Results

### 3.1 Bioinformatic Studies and Vector Construction

**FIGURE 1:**
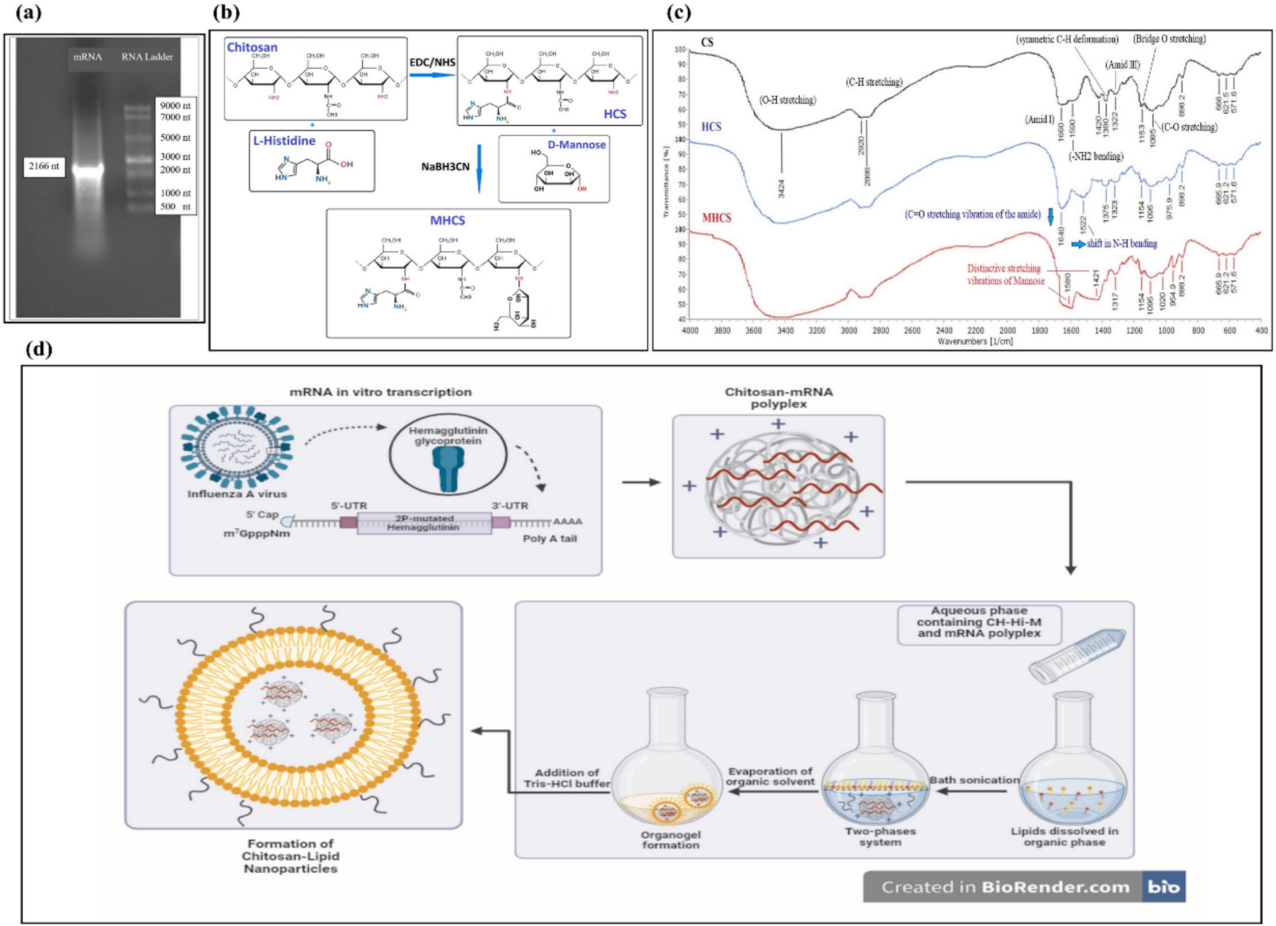
mRNA‐MHCS‐LNP vaccine preparation. (a) Agarose gel electrophoresis showing the size and integrity of the naked mRNA. (b) Reaction scheme for MHCS preparation, HCS: histidine‐conjugated chitosan, and MHCS: Mannose‐histidine‐conjugated chitosan. (c) FTIR spectra of CS, HCS, and MHCS. (d) The schematic shows the mRNA‐MHCS‐LNP vaccine design.

The codon‐optimized full‐length sequence of the influenza A (H1N1) hemagglutinin glycoprotein‐encoding gene, which contains two proline mutations (V399P‐S415P), is flanked by an optimized 5′UTR (from the human alpha‐globin gene) plus an optimized Kozak sequence and an efficient 3′‐UTR (from the human AES/TLE5 gene), followed by two termination codons and 110 adenine nucleotides as the poly(A) tail. mRNA in vitro was transcribed using T7 RNA polymerase and the modified nucleoside N1‐methyl pseudouridine and analyzed by denaturing gel electrophoresis, which was consistent with its calculated length of 2166 ribonucleotides, indicating purity and integrity (Figure 1a).

### 3.2 Synthesis and Characterization of HCSs and MHCSs

Histidine was conjugated to chitosan (CS) via a coupling reaction mediated by 1‐ethyl‐3‐(3‐dimethylaminopropyl)carbodiimide (EDC)/N‐hydroxysuccinimide (NHS), leading to the synthesis of histidine‐conjugated chitosan (HCS) polymers. Initially, EDC activated the carboxyl group on histidine, which subsequently reacted with the amino group at the C2 position of chitosan via NHS, leading to the formation of acylation polymers. Subsequently, the modification of the HCS polymer with mannose commenced with the ring opening of mannose, followed by reductive amination of the resulting aldehyde with the free amino groups present on the HCS polymer. The synthetic scheme depicting the mannose and histidine‐grafted chitosan is presented in Figure 1b. The synthesis of the conjugates was verified by the Fourier transform infrared spectroscopy (FTIR). Figure 1c displays the FTIR spectra of chitosan, histidine‐grafted chitosan, and mannose‐histidine‐grafted chitosan. The FTIR spectral analysis of chitosan indicated the presence of the following peaks. The broad peak at 3420 cm^−1^ corresponds to the stretching vibrations of OH, and the peaks at 2920 and 2888 cm^−1^ are associated with the aliphatic vibrations of CH stretching in the ‐CH and ‐CH2 groups. The amide frequencies include the N‐H bond stretching of amide I and N‐H straining vibrations of ‐NH2 groups, which are observed at 1660 and 1590 cm^−1^, respectively. The peak at 1380 cm^−1^ corresponds to the symmetric deformation of C‐H in the ‐CH3 group. Additionally, the 1322‐cm^−1^ peak is attributed to the vibration modes of amide III. Furthermore, the absorption band at 1153 cm^−1^ corresponds to the antisymmetric stretching of the C‐O‐C bridge, while the stretching vibration at 1085 cm^−1^ is attributed to the C‐O stretching vibration of alcohol groups. Following the conjugation of histidine, an enhancement in the peak at 1640 cm^−1^, which is associated with the C=O stretching vibration of the amide, and a shift in the peak corresponding to the N–H bending vibration from 1590 to 1522 cm^−1^, verified the successful attachment of histidine to the CS backbone via an amide bond. Moreover, two stretching vibrations distinctive of mannose are indicated by absorption peaks in the 1580 and 1421 cm^−1^ regions. The degree of substitution (DS) of the glucosamine residues in the chitosan for each prepared polymer was calculated by assessing the increase in the amide I peak (1660 cm^−1^) following modification, utilizing the peak of O–H stretching at 3422 cm^−1^ as an internal reference, as outlined below (Equation 1, Equation 2) [20]:

(1) $$\mathrm{DS}\;\left(\%\right)=\frac{{\left(\frac{T_{1660}}{T_{3422}}\right)}_{\mathrm{CH}-\mathrm{Hi}}-{\left(\frac{T_{1660}}{T_{3422}}\right)}_{\mathrm{CH}}}{\left(1-0.164\right)}\times 100$$

(2) $$\mathrm{DS}\;\left(\%\right)=\frac{{\left(\frac{T_{1660}}{T_{3422}}\right)}_{\mathrm{CH}-\mathrm{Hi}-\mathrm{Man}}-{\left(\frac{T_{1660}}{T_{3422}}\right)}_{\mathrm{CH}-\mathrm{Hi}}}{\left(1-0.164\right)}\times 100$$

*T*_1660_ and *T*_3422_ indicate the peak heights calculated using a baseline set between 800–1900 and 1900–3850 cm^−1^, respectively. The DS outcomes are displayed in Table 1.

**TABLE 1:** The degree of L‐histidine and D‐mannose substitution of the modified polymers was determined through FTIR.

Polymer	DS (%) theoretical^a^	DS (%)
HCS	28.5%	21%
MHCS	25%	9.7%

^a^ Determined by the ratio of moles of L‐histidine and D‐mannose to moles of charged glucosamine residue (0.6 g of L‐histidine monohydrochloride = 3.538 mmol/2 g of chitosan = 12.42 mmol of glucosamine).

### 3.3 Synthesis of mRNA‐MHCS‐LNPs

Chitosan‐LNPs were formulated using phosphatidylcholine and cholesterol as the core and a shell consisting of mannose‐histidine‐conjugated chitosan in a reverse‐phase evaporation process (Figure 1d).

**FIGURE 2:**
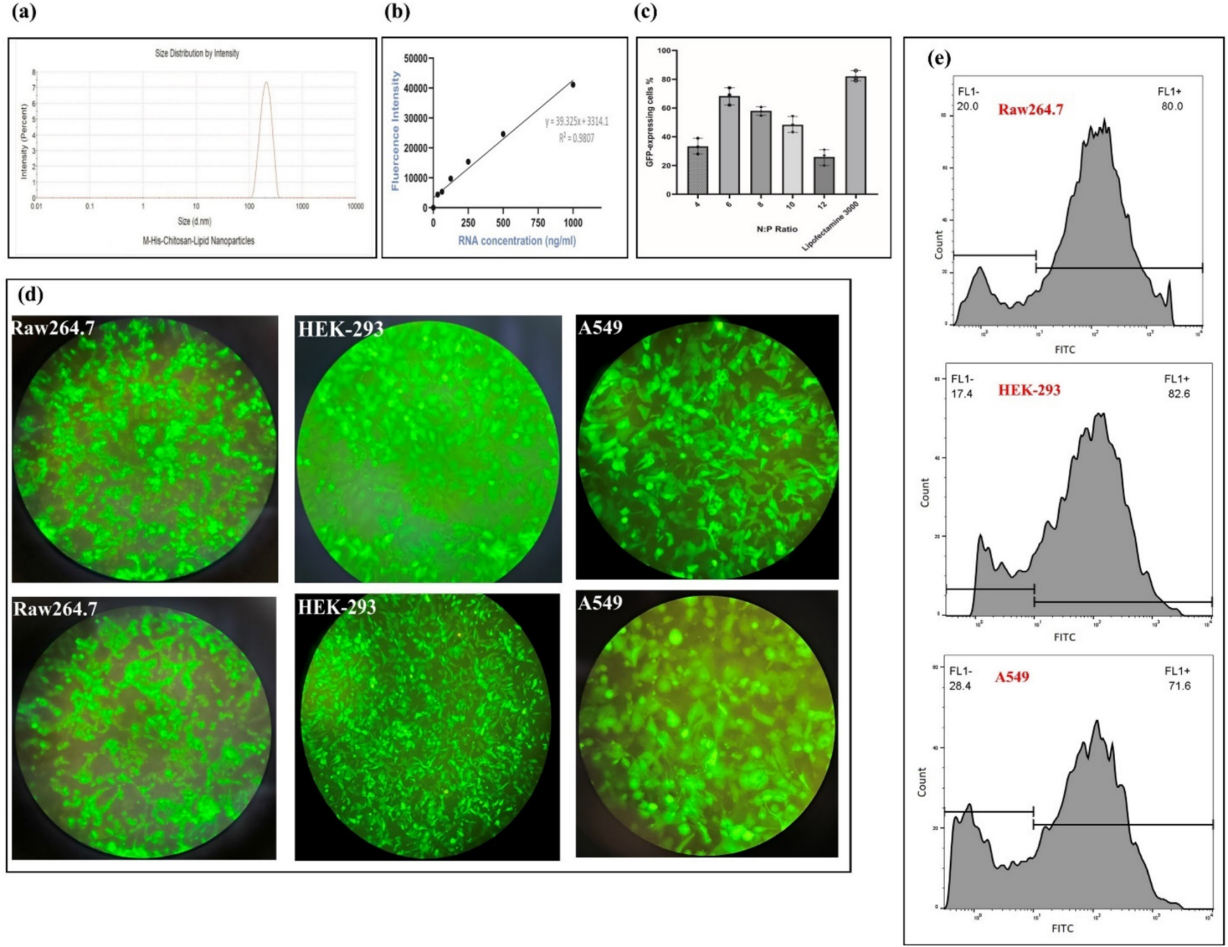
Characterization of the mRNA‐MHCS‐LNPs. (a) DLS results representing hydrodynamic diameter vs. intensity. (b) The standard curve shows a linear relationship between the RNA concentration and fluorescence intensity. (c) mRNA transfection assays. Five N:P ratios (4, 6, 8,10, and 12) of MHCS‐LNPs with GFP‐expressing mRNA (at a concentration of 1 μg) were used. Transfection efficiency was calculated as the proportion of cells expressing GFP relative to the total number of cells counted via flow cytometry. The data are shown as the mean ± standard deviation (*n* = 3). (d) The transfection efficiency of the GFP‐encoding mRNA‐MHCH‐LNPs in three cell lines, namely, RAW264.7, HEK293, and A549, was investigated using fluorescence microscopy and (e) flow cytometry analysis.

For a comprehensive structural characterization of mRNA‐MHCS‐LNPs, assessing their size and surface charge is necessary. Determining the hydrodynamic diameter is especially critical for colloidal particles, as it facilitates further studies and applications. DLS is the most common technique employed to ascertain the size distribution of small particles within a solution or suspension. This method involves measuring the variations in scattered light intensity over time, induced by the particles' Brownian motion [49]. According to the DLS results, the nanoparticles exhibited a hydrodynamic diameter of 200 nm (CV% = 8.62%). Additionally, the polydispersity index demonstrated a permissible value of 0.05, confirming the uniformity of the prepared mRNA‐MHCS‐LNPs (Figure 2a). The single peak observed in the DLS results, particularly in terms of intensity, signifies the lack of nanoparticle aggregation. The zeta potential was measured at +29.4 mV.

### 3.4 Encapsulation Efficiency

To assess the effectiveness of MHCS‐LNPs in mRNA encapsulation, a quantitative fluorescence spectroscopy method was used, as previously described. This fluorescence‐based assay employs a dye, SYBR Green II, that is, nonfluorescent in solution but becomes fluorescent when it binds to nucleic acids. A calibration curve was created by serially diluting the RNA standard, as detailed in Section 2. The fluorescence intensities of each RNA concentration were measured after adding the SYBR Green II dye to establish a calibration curve that correlates fluorescence intensity with RNA concentration. The standard curve demonstrated an excellent linear relationship (*R* ^2^ = 0.980) between the RNA concentrations and fluorescence intensity. This relationship was then used to estimate the encapsulation efficiency. Triton X‐100 was added to the mRNA‐MHCS‐LNPs sample to disrupt the particles, allowing the detection of the total mRNA present in the well. The difference between the total mRNA sample (with Triton X‐100) and the free, nonencapsulated mRNA is used to calculate the concentration of encapsulated mRNA and the encapsulation efficiency. The encapsulation efficiency should generally exceed 80%. MHCS‐LNPs encapsulation efficiency was determined to be approximately 95% (refer to Figure 2b).

(3) $$\%\mathrm{EE}=\frac{231.12\mathrm{ug}-9.53\mathrm{ug}}{231.12\mathrm{ug}}\times 100=\%95$$

### 3.5 In Vitro Transfection Efficiency

To utilize a gene nanocarrier either in vitro or in vivo, it is crucial to assess its transfection efficiency and potential toxicity. The N:P ratio represents the ratio between the amine groups (N) of the modified chitosan and the phosphate groups (P) of the mRNA (one per base for RNA and two for DNA) and significantly impacts nanoparticle properties such as complexation efficiency, particle size and stability, and transfection efficiency, which a well‐balanced N:P ratio ensures that nanoparticles can effectively protect nucleic acids from degradation and facilitate their entry into cells, cellular uptake and endosomal escape, and toxicity and immunogenicity. A higher N:P ratio typically results in smaller nanoparticles. This occurs because an increased ratio of amine groups to phosphate groups of the nucleic acids strengthens electrostatic interactions, leading to the formation of more compact and stable complexes. Small‐sized nanoparticles (∼100 nm) have been found to exhibit more than three times greater cellular uptake compared to larger nanoparticles (∼275 nm). Also, transfection efficiency depends on several parameters, with one of the most important being the size of the particles used to form polymer/mRNA complexes. Several studies, some of which contradict each other, have been conducted to investigate the effect of size on the efficiency of nanoparticle‐mediated transfection. Larger nanoparticles (200–500 nm) showed higher transfection efficacy compared to that of the smaller size (40–100 nm). This is due to several factors: they settle onto the cell surface more quickly than smaller particles, increasing contact with the cells and promoting cellular internalization; they contain a larger proportion of free cationic polymers in addition to those complexed with DNA, which helps destabilize the membrane and facilitate entry into cells; and their endosomolytic activity is significantly higher than that of smaller particles [50]. On the other hand, some studies show that since smaller nanoparticles have a larger surface area and have a higher percentage of molecules exposed on the particle surface to interact with cells, this can lead to enhanced cellular uptake but also higher cytotoxicity. Overall, the optimization of the N:P ratio in nanoparticles should be tailored based on the specific application, the route of administration, and safety requirements [51]. The HEK293T cell line was selected for these evaluations due to its widespread use in transfection experiments and the production of recombinant proteins. For transfection efficiency analysis, HEK293 cells were seeded in 24‐well plates at a density of 5 × 10^4^ cells per well overnight. After 48 h, the transfection of MHCS‐LNPs carrying 1 μg of mRNA with different N:P ratios (from 4 to 12) were examined using a fluorescence microscope to differentiate transfected cells through GFP expression from untransfected cells. Additionally, transfection was performed using Lipofectamine 3000 reagent as a positive control, adhering to the standard protocols supplied by the respective manufacturers. As shown in Figure 2c, the most efficient formulation for mRNA transfection was at N:P ratio of 6.

After determining the best N:P ratio, the expression of GFP was explored in three different cell lines (HEK 293T, A549, and RAW264.7) following transfection with MHCS‐LNPs encapsulating 1 μg of GFP‐encoding mRNA. The fluorescence microscopy and flow cytometry results revealed that approximately 71.6% to 82.6% of the cell population in all three cell types studied expressed GFP (Figure 2d). This indicates the effectiveness of mRNA‐MHCS‐LNPs in overcoming various barriers to successfully delivering and translating the enclosed mRNA.

### 3.6 mRNA‐MHCS‐LNPs Stability and Storability

**FIGURE 3:**
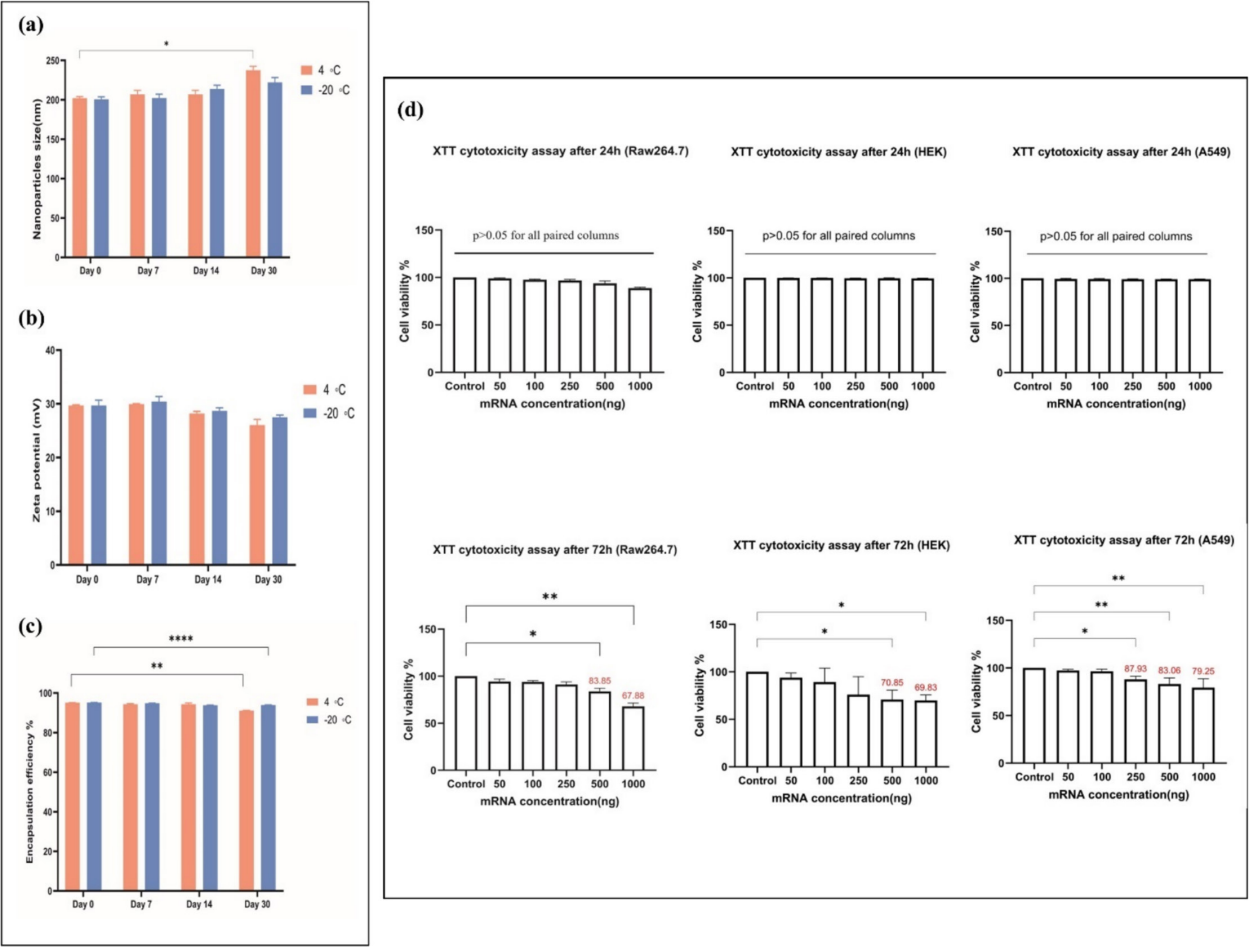
mRNA‐MHCS‐LNP stability and storability were evaluated. (a) Analysis of mRNA‐MHCS‐LNP size by DLS revealing the Z‐average sizes (nm) of the mRNA‐MHCS‐LNPs kept at 4°C or −20°C for the specified durations. (b) Analysis of zeta potentials (in mV) of mRNA‐MHCS‐LNPs kept at 4°C or −20°C for the specified time points determined by a zeta‐sizer. (c) Analysis of the encapsulation efficiency (expressed as a percentage) of mRNA‐MHCS‐LNPs kept at 4°C or −20°C, as determined by a fluorescence‐based assay. The height of the bars indicates the mean ± SD, and statistical significance was determined by two‐way ANOVA. *p ≤ 0.05, **p < 0.01, ***p < 0.001, ****p < 0.0001. (d) Assessment of the cytotoxicity of mRNA‐MHCS‐LNPs utilizing the XTT viability assay. HEK293T, RAW264.7, and A549 cells were exposed to varying quantities of mRNA‐MHCS‐LNPs for 24 and 72 h. The data are the means ± SDs of one experiment performed in triplicate, and statistical significance was calculated by one‐way ANOVA. *p ≤ 0.05, **p < 0.01, ***p < 0.001, ****p < 0.0001.

DLS and a zeta‐sizer were used to examine the changes in the size and zeta potential of the nanoparticles. Figure 3a–c shows the evolution trends in the mean particle size, zeta potential, and encapsulation efficiency throughout an extended storage duration from Day 0 to Day 30 at two different storage temperatures. The resulting data indicate a slight (*p* > 0.05, two‐way ANOVA) increase in the mean particle size of the mRNA‐MHCS‐LNPs from 202 and 200.6 nm (Day 0) to 206.83 and 202.16 nm (Day 7) after storage at 4°C and −20°C, respectively, as shown in Figure 3a. Also, the mean particle size after storage at 4°C showed a minor increase (*p* > 0.05, two‐way ANOVA) from Day 7 (206.8 nm) to Day 14 (210 nm). A significant rise in nanoparticle size (*p* < 0.05, two‐way ANOVA) was observed on Day 30 (237.5 nm) after storage at 4°C. However, nanoparticles stored at −20°C showed only a minor increase (*p* > 0.05, two‐way ANOVA) in the average diameter from Day 0 to Day 30. The significant rise in mRNA‐MHCS‐LNP size may be linked to the development of particle agglomerates during storage in storage buffer (pH 5.5) and a change in pH at room temperature.

Conversely, the zeta potential of the mRNA‐MHCS‐LNPs stored at 4°C remained unchanged (*p* > 0.05, two‐way ANOVA) from Day 0 to Day 14, as depicted in Figure 3b. A minor (*p* > 0.05, two‐way ANOVA) decrease in the zeta potential from +29.7 to +26.05 mV was observed from Day 0 to Day 30. Furthermore, the results indicated no substantial alteration in the zeta potential of the mRNA‐MHCS‐LNPs stored at −20°C throughout the entire storage period.

The mRNA‐MHCS‐LNPs stored at 4°C showed a moderately significant (*p* < 0.01, two‐way ANOVA) decrease in encapsulation efficiency from Day 0 (95.13%) to Day 30 (91.15%), and the mRNA‐MHCS‐LNPs stored at −20°C showed no significant mRNA leakage during the storage period (Figure 3c).

Collectively, these data highlight the physicochemical properties and stability of our mRNA‐MHCS‐LNPs. Our findings indicate that while refrigerator storage temperature leads to an increase in nanoparticle size and a decrease in encapsulation efficiency within 30 days, the overall adverse alteration remains below 10% in comparison to their value just after formulation. The mRNA‐MHCS‐LNP formulations retained acceptable particle size, zeta potential, and encapsulation efficiency.

### 3.7 In Vitro Cytotoxicity of the mRNA‐MHCS‐LNPs

While it is preferable for the particle surface to have a high positive charge density to ensure efficient mRNA complexation, highly cationic particles often exhibit significant cytotoxicity. Therefore, a balance must be struck between these two factors. The XTT assay is a quantitative method used to assess cell proliferation rates under various conditions and to evaluate the toxicity levels of these conditions on the cells. This method relies on the reduction of XTT, a tetrazolium salt, into orange formazan crystals by metabolically active cells. In this study, the cytotoxicity effect of mRNA‐MHCS‐LNPs was assessed via the XTT method on the HEK‐293T, A549, and RAW264.7 cell lines, and the relative viability of cells at 24‐ and 72‐h postexposure was calculated. After 24 h of consistent treatment with the mRNA‐MHCS‐LNPs, the viability of RAW264.7, A549, and HEK293T cells did not significantly change (*p* > 0.05). After 72 h, the cell viability of RAW264.7 cells moderately decreased (*p* ≤ 0.01) at the maximum concentration of mRNA (1000 ng), and the mRNA exhibited no considerable cytotoxic effect on the cells, particularly at higher concentrations (500 and 1000 ng). As the mRNA concentration increases, the percentage of viable cells decreases. After 72 h, A549 cells showed a moderate decrease (*p* ≤ 0.01) in viability when exposed to mRNA‐MHCS‐LNPs containing ≥ 0.25 μg of mRNA (corresponding to ≥ 0.78 μg of mRNA per square centimeter). Likewise, the viability of HEK293T cells slightly diminished (*p* ≤ 0.05) after 72 h of exposure to nanoparticles containing ≥ 0.5 μg of mRNA (equivalent to ≥ 1.56 μg of mRNA per square centimeter). Nevertheless, even after 72 h of sustained treatment with the highest amounts of mRNA‐MHCS‐LNPs encapsulated 1 μg of mRNA, the viability stayed above 79% for A549 cells, above 68% for RAW264.7 cells, and above 69% for HEK293T cells (refer to Figure 3d).

### 3.8 Animal Studies

**FIGURE 4:**
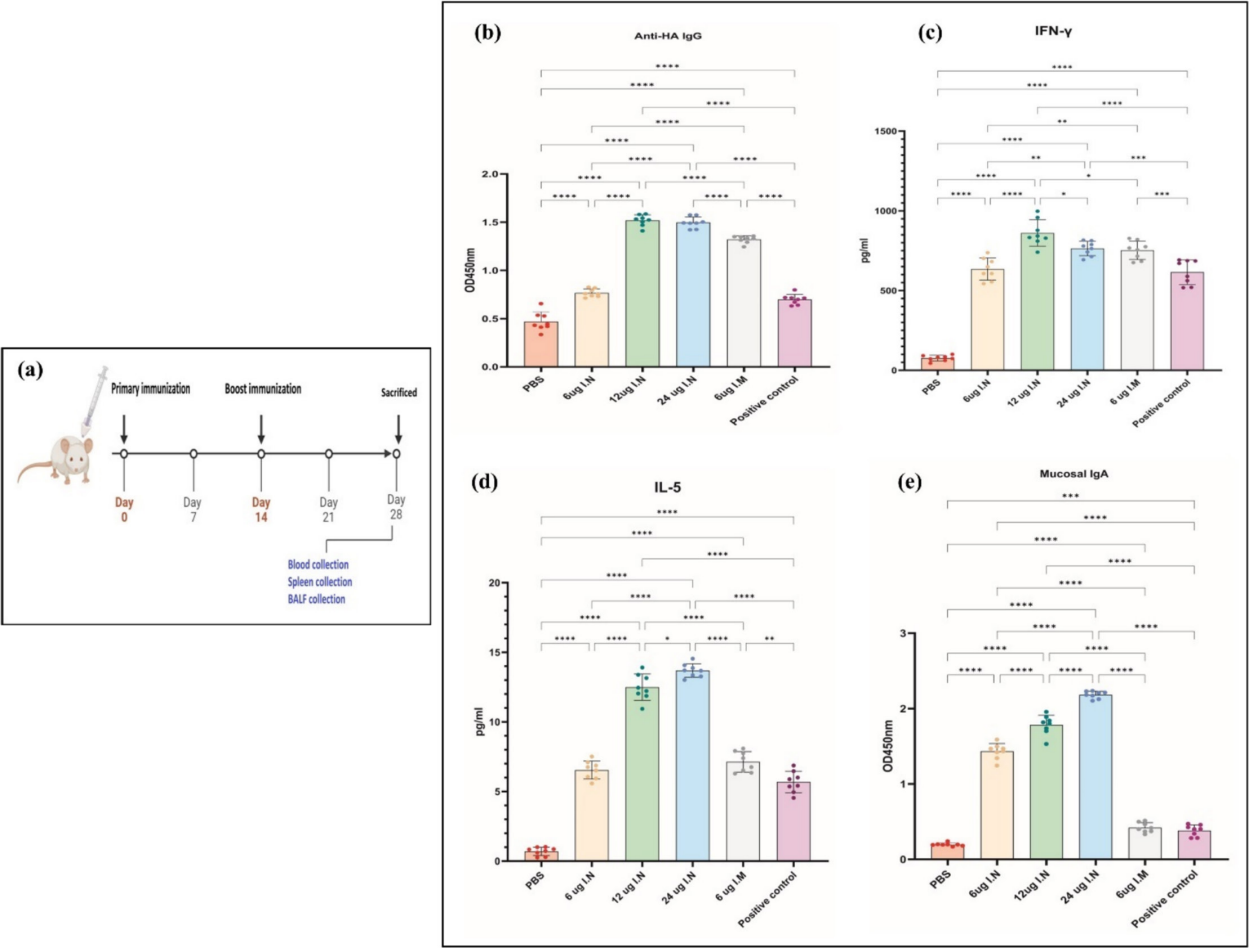
(a) Overview of the vaccination plan and sample collection of animals to analyze the immunogenicity and safety of the mRNA‐MHCS‐LNP vaccine. (b) HA‐specific serum‐bound IgG after vaccination with HA‐encoding mRNA‐MHCS‐LNPs in mice. Female BALB/c mice were divided into six groups (*n* = 8 per group) and immunized intranasally (6, 12, or 24 μg of mRNA) or intramuscularly (6 μg of mRNA) with the HA‐encoding mRNA‐MHCS‐LNP vaccine, PBS, or commercial influenza vaccine (as a positive control group). The titer of HA‐specific IgG antibodies in the mice's serum at a 1:1000 dilution was measured using an ELISA kit. (c, d) T‐cell immune response in mRNA‐MHCS‐LNPs‐immunized mice. The concentrations of the secreted Th2 cytokine IL‐5 and Th1 cytokine IFN‐γ were quantified using ELISA. (e) Mucosal IgA response in mRNA‐MHCS‐LNP‐vaccinated mice. The mice were euthanized on the 28th day after the first immunization, and the BALF samples were collected. The levels of the secreted mucosal IgA were measured by ELISA. Each data point corresponds to an individual mouse. Statistical analysis was conducted using GraphPad Prism 10 software. The data are presented as the mean ± standard error of the mean. Statistically significant differences were calculated using one‐way ANOVA (significant differences are shown as ns: *p* > 0.05, *: *p* < 0.05, **: *p* < 0.01, ***: *p* < 0.001, ****: *p* < 0.0001).

To evaluate the immunogenicity of the mRNA‐MHCS‐LNPs, six‐ to eight‐week‐old female BALB/c mice were divided into six groups and inoculated twice, on Day 0 and Day 14. Two doses of nanoparticles containing 6, 12, or 24 μg of mRNA were administered intranasally. One other group received a low‐dose mRNA (6 μg) via the intramuscular route, a negative control group received PBS intranasally, and a positive control group received a commercial influenza vaccine (VaxigripTetra) via the intramuscular route (Figure 4a). VaxigripTetra is an inactivated quadrivalent nonadjuvanted influenza vaccine of the following strains: A/Victoria/4897/2022 (H1N1)pdm09‐like strain, A/Darwin/9/2021 (H3N2)‐like strain, B/Austria/1359417/2021‐like strain, and B/Phuket/3073/2013‐like strain. Each vial contains 15 μg of hemagglutinin glycoprotein per strain (totaling 60 μg). To adjust the human dose for BALB/c mice, which typically weigh around 20 g, the required dose for each vaccination was calculated 0.25 μg of hemagglutinin glycoprotein [52]. During the immunization period, no significant changes in mice body weight were detected, and no deaths were reported after 1 month in any of the groups (data not shown).

### 3.9 Evaluation of Humoral Immune Responses

The effectiveness of the formulated HA‐encoding mRNA‐MHCS‐LNPs in stimulating a robust humoral immune response was examined using an ELISA method (Figure 4b). The findings showed a notable increase in the level of anti‐HA IgG antibodies in the serum of mice that received mRNA compared with that in the PBS group. Increasing the intranasal dose from 6 to 12 μg significantly (*p* ≤ 0.0001) increases the IgG response. However, increasing the dose further to 24‐μg intranasal does not significantly (*p* > 0.05) increase the IgG response compared to the 12‐μg dose. This suggests a potential plateau in the dose‐response relationship for intranasal administration. Mice that received 12 μg of the mRNA‐MHCS‐LNPs showed elevated levels of HA‐specific IgG compared to the mice immunized via intramuscular (*p* ≤ 0.0001) (6 μg mRNA‐MHCS‐LNPs) and positive control (intramuscular injection of the commercial influenza vaccine VAXIGRIP) routes. Notably, the low‐dose intranasal group had slightly greater responses than the positive control group (*p* > 0.05), but at the 6‐μg dose, intramuscular administration results in a higher IgG response than intranasal administration. Therefore, these findings indicate that two doses of the HA‐encoding mRNA‐MHCS‐LNP vaccine prompt significant increases in the levels of anti‐HA IgG antibodies in mice.

### 3.10 Investigation of the Cell‐Mediated Immune Response

Spleen tissues were harvested from BALB/c mice that had been administered mRNA‐MHCS‐LNPs at doses of 6, 12, or 24 μg via the intranasal route or 6 μg via the intramuscular route, containing HA‐encoding mRNA, on Day 28 following booster vaccination to explore the cell‐mediated immune response. After stimulation of cells with a peptide pool containing influenza A hemagglutinin, the secretion of FN‐γ and IL‐5 from mice was measured by ELISA kits. As demonstrated in Figure 4b, the findings showed strong secretion of the cytokine IFN‐γ on Day 28 postinitial vaccination, peaking at approximately 900 pg ml^−1^. The concentration of IFN‐γ in the supernatants of splenocyte cultures was greater in mice vaccinated with 12 μg of mRNA‐MHCS‐LNPs than in all other groups (*p* ≤ 0.0001). This implies that the mRNA‐MHCS‐LNPs vaccine at this concentration induced a stronger cell‐mediated immune response, as IFN‐γ is a key cytokine in the Th1 response. In the same doses (6‐μg mRNA) but different administration routes, the intramuscular route presents higher levels (*p* ≤ 0.01), though slightly lower than the 12 μg intranasal group, of IFN‐γ production. Increasing the intranasal dose from 6 to 12 μg significantly (*p* ≤ 0.0001) increases the IFN‐γ response. However, increasing the dose further to 24‐μg intranasal appears to slightly reduce the IFN‐γ response compared to the 12‐μg dose. Additionally, there was no discernible difference (*p* ≤ 0.05) between the 12 μg, and 24 μg of intranasal groups and 6‐μg intramuscular groups.

Moreover, splenocytes were assessed for the production of the cytokine IL‐5, which is induced in the Th2 T‐cell response. Since IL‐5 prompts antigen‐activated B cells to mature into immunoglobulin‐secreting plasma cells and boosts IgA secretion, elevated levels of IL‐5, peaking at approximately 15 pg ml^−1^ in intranasally vaccinated mice compared to those in intramuscularly vaccinated and control groups, represent robust mucosal immune responses. Increasing the intranasal dose from 6 to 12 μg leads to a significant enhancement (*p* ≤ 0.0001) in the IL‐5 response. Raising the dose further to 24 μg via intranasal administration also boosts the IL‐5 response when compared to the 6‐μg dose (*p* ≤ 0.0001); however, this increase is not observed when compared to the 1‐μg dose (*p* ≤ 0.05). At the 6‐μg dose, intramuscular administration and intranasal administration resulted in similar IL‐5 levels, and the 24‐μg intranasal administration provided the best IL‐5 production.

### 3.11 Evaluation of Mucosal IgA Responses

To explore the stimulation of mucosal immunity induced by the mRNA‐MHCS‐LNP nanoparticles in mice, the existence of HA‐specific IgA antibodies in the BALF samples was assessed at 28 days following the initial immunization. The findings indicated that IgA levels in the BALF samples of all intranasal vaccine groups were significantly higher than those in the PBS or commercial flu vaccine groups (Figure 4e). The highest level of the BALF IgA was detected in the intranasal high‐dose group (24‐μg mRNA), which is consistent with previous findings and also indicates that the level of the mucosal IgA antibody increases in a dose‐dependent manner and have significant differences (*p* ≤ 0.0001) with all other groups. Intramuscular immunization did not lead to notable IgA responses in any of the mucosal samples examined. These results indicated that antigen‐specific IgA response in BALF samples was successfully elicited by the mRNA‐MHCS‐LNP nanoparticles via the intranasal route in mice, and mucosal IgA response is strongly dose‐dependent with intranasal administration, peaking at 24‐μg mRNA, and the 6‐μg intramuscular dose results in the lowest IgA levels among the experimental conditions. The results highlight that intranasal administration of the vaccine produces a superior immune response at both the 12‐ and 24‐μg doses when compared to intramuscular administration.

### 3.12 Hemagglutination Inhibition (HAI) Antibodies

**FIGURE 5:**
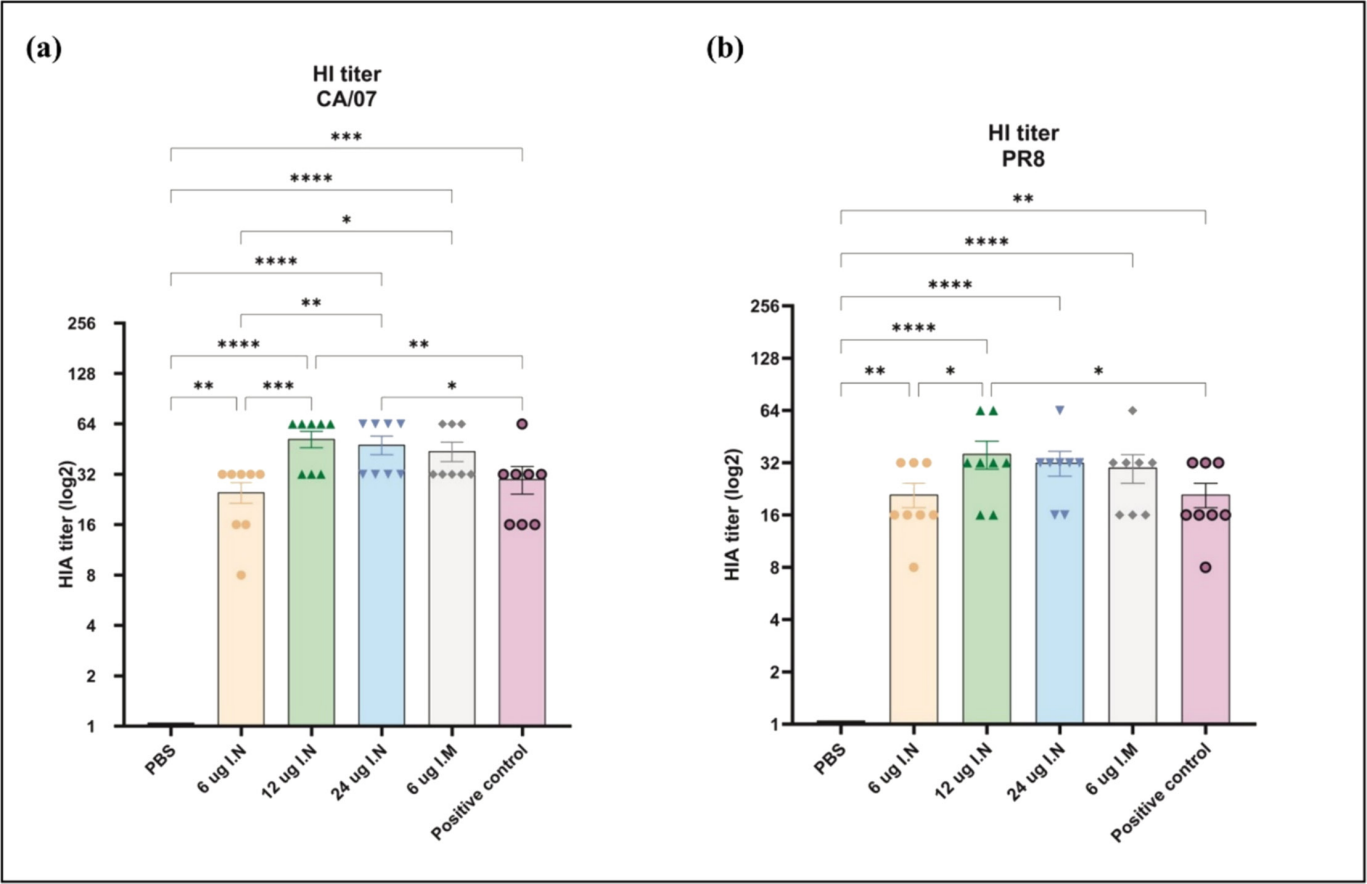
Hemagglutination inhibition antibodies assay (a) against influenza A H1N1/CA/07 and (b) against influenza A H1N1/PR8‐specific serum HAI titers. The bars indicate the GMT serum titer, and the error bars represent the 95% confidence intervals for each study group. The data were analyzed using a nonparametric Kruskal–Wallis test. *p < 0.05; **p < 0.01; ***p < 0.001; ****p < 0.0001.

Blood samples were taken on Day 28 after the initial vaccination, and 1:10 dilutions of the serum samples were tested for receptor‐blocking antibodies using an HAI assay against the influenza A reference strains A/California/07/2009 (H1N1) (also known as CA/07) and A/Puerto Rico/8/34 (H1N1) (PR8) viruses. The serum HI titers (Figure 5) closely mirrored the serum antibody responses. Intranasal administration of 12 and 24 μg of mRNA‐MHCS‐LNPs led to HI titers comparable to (*p* > 0.05) and frequently greater than those attained through 6‐μg intramuscular immunization and positive control groups. Like previous findings, the HI titers of the medium‐dose intranasal group treated with 12 μg of mRNA had the highest mean among all the groups tested, consistently surpassing the HI titers of the 6‐μg intramuscular group. However, equivalent doses (6‐μg mRNA) administered via different routes showed significant differences (*p* ≤ 0.05), with geometric mean HI titers of 226.2 for the intranasal group and 415 for the intramuscular group. Intranasal administration of 12 μg of mRNA results in an HI titer approximately twice that of the lowest mRNA dose (6 μg); but consistent with the HA‐specific IgG data, the HI titers also indicated that increasing the mRNA dose from 12 μg (493) to 24 μg (452) did not result in an increase in neutralizing antibody levels (*p* > 0.05). This may occur when the immune system reaches a saturation point where further increases in antigen concentration do not proportionally increase antibody production. This suggests that the median vaccine dose (12‐μg mRNA) is effective and comparable (*p* > 0.05) to the optimal intramuscular dose (6‐μg mRNA).

The highest HI titers against the H1N1/PR8 virus were observed in the 12‐ and 24‐μg intranasal groups, with geometric mean HI titers of 320 and 293, respectively (Figure 5b). These values were slightly greater than those of the intramuscular group, with a mean HI of 270. As shown in Figure 5a, the HI titers against the H1N1‐CA/07 virus were slightly greater than those against the H1N1/PR8 virus in all the tested groups. The highest titers were noted with geometric mean HI titers of 493 and 452 for the 12‐ and 24‐μg intranasal groups, respectively. Given that the mRNA construct for the vaccine antigen was designed based on the CA/07 hemagglutinin protein, it is reasonable to expect an increase in neutralizing antibody levels compared to those of the hemagglutinin protein of the H1N1/PR8 virus.

## 4 Discussion

During the COVID‐19 pandemic, there was a significant clinical requirement to halt the transmission chain, reduce breakthrough infections, and attain lasting elevated levels of protection against severe forms of COVID‐19. This has highlighted the potential of nasal vaccines, which are appealing for establishing mucosal immunity and enhancing and probably strengthening the systemic immunity acquired through intramuscular injections. A recent publication by Tang et al. [53] illuminated the limitations of intramuscular mRNA vaccines in generating respiratory mucosal immunity against Omicron in individuals while demonstrating the success of achieving this effect with a nasal vaccine in mice. In this research, 19 vaccinated individuals were contrasted with 10 who had recovered from COVID‐19 and 5 who had not been vaccinated. Despite comparable levels of circulating neutralizing antibodies against the D614G, Delta, and Omicron variants, the vaccinated group exhibited significantly lower neutralizing titers against all variants in bronchoalveolar lavage (BAL) fluid than did the convalescent group. Additionally, the BAL tissue of the vaccinated group displayed notably fewer spike‐specific memory CD4 T cells, CD8 T cells, and RBD‐specific memory B cells than that of the group with a history of COVID‐19 [53].

FluMist (MedImmune, LLC) is the first live‐attenuated influenza virus (LAIV) nasal vaccine that has received successful approval and commercialization in the United States and Europe [54]. FluMist is a cold‐adapted LAIV vaccine, and studies have shown that it is more effective than inactivated influenza vaccines alone (∼50%–60%) and that it offers cross‐protective immunity [55]. However, safety issues regarding FluMist have been raised, particularly in vulnerable groups such as infants under 2‐years old and individuals over 50‐years old. FluMist and other LAIVs have the potential to undergo genetic reassortment and revert to a more virulent form, presenting a risk. In addition, due to the recent reports of inadequate protection against the H1N1pdm09 component in children aged 2–17 years, the US Centers for Disease Control and Prevention (CDC) withdrew its endorsement for the utilization of LAIV in the United States during the 2015–2016 and 2016–2017 seasons. In this regard, there is an urgent need for new, virus‐independent, safe, and effective nasal influenza vaccines that can provide broader cross‐protection with high efficacy.

Nonetheless, creating vaccines for intranasal use poses significant challenges. The respiratory system is safeguarded by a mildly acidic mucosal layer with proteolytic enzymes, creating a barrier to the epithelial cells that are consistently cleared. While these defenses protect against respiratory pathogens, they can hinder antigen delivery via intranasal vaccination [56].

Overcoming the challenges of intranasal vaccination requires an efficient, nontoxic, and site‐specific delivery system. As an antigen delivery system, mannosylated chitosan nanoparticles improve nasal immunization by enhancing the retention of antigens in the nasal cavity and enabling controlled, sustained release to stimulate stronger and longer‐lasting immune activity. Mannose is currently the only glycotrophic nutrient used in clinical applications, and mannosylated carrier systems can be recognized and internalized by cells through receptor‐mediated endocytosis. This targeting is achieved by binding to the mannose receptor (MR) expressed on APCs. The internalized antigens are then processed and presented through both the MHC class I and MHC class II pathways. This helps to bolster overall immune activity, including the activation of cytotoxic T cells and other cell‐mediated immune mechanisms [25]. Despite all the desirable specifications of mannosylated chitosan, the transfection effectiveness of chitosan as a carrier is limited, partly due to its inadequate endosomal escape rates. This deficiency primarily stems from the weak buffering capacity of chitosan, which hinders its ability to facilitate endosomal/lysosomal escape. The transfection efficiency of mannosylated chitosan nanoparticles encapsulating GFP‐coding DNA, based on flow cytometry data, was extremely poor. Grafting an imidazole ring, as a part of the histidine amino acid, to the chitosan backbone significantly increased the polymer transfection efficiency. The positive charge of the primary amino group in histidine enables it to bind to nucleic acids, and as a result of increasing chitosan buffering capacity in the pH range of endosomes and lysosomes, neutralization of the low pH enhances endosomal escape. However, MHCS nanoparticles are not efficient enough for use as gene delivery systems. Finally, the synthesis of chitosan‐LNP hybrid systems combined with both favorable characteristics of lipid‐based delivery systems and polymers showed promising transfection efficiency in all three transfected cell lines.

Among all the physiological properties of nanoparticles, size has the greatest impact on vaccine immunogenicity. Studies showed that when nanoparticle size increases from 30 to 200 nm, the antigen is more efficiently delivered into the MHC class II presentation pathway, thereby enhancing the proliferation of antigen‐specific CD4+ T cells. The increased immunogenicity of the bigger nanoparticles may be partially attributed to their efficient uptake by APCs. The cellular uptake of various antigen‐delivery systems is size‐dependent in both rodent macrophages and human DCs [57]. Also, nanoparticles size of 200 nm are similar in size to most respiratory viruses and mimic the natural structure of viruses to raise humoral and cellular immune responses more efficiently. The size of the vaccine nanoparticles also affects the kinetics of drainage into lymph nodes. The larger the nanoparticle, the slower its diffusion into lymph nodes. According to most studies, nanoparticles with a size of approximately 200 nm are internalized by immune cells through clathrin‐dependent endocytosis and elicit a balanced Th1 and Th2 immune response [58, 59]. One considerable point in this regard is that, despite the numerous advantages of intranasal nanoparticle vaccine for delivering immunogens directly to the mucosal surfaces, this route also raises concerns regarding the risk of unwanted penetration through the blood–brain barrier (BBB). Studies showed that when the nanoparticle size is below 200 nm, they are more likely to interact with neurons in the nasal mucosa and migrate into the central nervous system (CNS) [60]. In this context, mRNA‐MHCS‐LNPs are designed to be sufficiently small to effectively penetrate mucosal surfaces while being large enough to prevent unwanted (CNS) penetration.

The durability and extended shelf life of the mRNA‐MHCS‐LNPs were assessed following 30 days of refrigeration (4°C) or storage at −20°C. DLS was used to track the changes in the hydrodynamic diameter and zeta potential of the nanoparticles over time, and the MHCS‐LNPs were deemed unstable if these parameters rose by over 10% from their initial values postformulation. The data demonstrated that mRNA‐MHCS‐LNPs maintain their acceptable particle size, encapsulation efficiency, and zeta potential when frozen at −20°C. mRNA‐MHCS‐LNPs maintained an acceptable size when stored at −20°C, with a hydrodynamic diameter increase of less than 10%. However, when refrigerated, the particle size increased by approximately 13%. The formulated MHCS‐LNPs are essentially lipid nanoemulsions that are susceptible to destabilization. Based on previous findings, adjusting the ratio of phospholipids to chitosan, the application of surfactants and stabilizers such as polyethylene glycol (PEG), the application of low‐molecular‐weight chitosan, freeze‐drying the nanoparticles, and a higher zeta potential provide more stable MHCS‐LNPs, which renders the vaccines ideal for transportation and potentially reduces the need for strict maintenance before administration [33].

The balance between ensuring efficient mRNA complexation and minimizing cytotoxicity is a critical consideration when designing nanoparticles for mRNA delivery. While a high positive charge density on the particle surface is desirable for efficient mRNA binding, the inherent cytotoxicity of highly cationic particles necessitates careful optimization. The observed cell viability, assessed across RAW264.7, A549, and HEK293T cell lines, revealed that the mRNA‐MHCS‐LNPs generally exhibit low cytotoxicity, particularly at lower mRNA concentrations and after a shorter exposure period of 24 h. However, a concentration‐dependent decrease in cell viability was observed after 72 h, suggesting a potential cumulative cytotoxic effect at higher mRNA concentrations. Higher doses of mRNA or lipids and polymers of the nanoparticles may cause overstimulation of immune responses or disrupt cellular processes, leading to apoptosis or necrosis. The intracellular accumulation of mRNA can overload cellular machinery, particularly the ribosomes and translation machinery. Different cell types exhibit varying degrees of sensitivity to mRNA‐induced toxicity, and immune cells may react more strongly to mRNA because of their role in immune surveillance.

Exploring the immunogenicity and protective efficacy of intranasally administered HA‐mRNA MHCS‐LNP vaccines in mice showed that two‐dose vaccination could induce the robust generation of HA‐specific IgG and HA‐neutralizing antibodies and the production of IFN‐γ and IL‐5 cytokines. Intranasal administration of the median dose (12 μg) of mRNA‐MHCS‐LNPs elicited systemic immune responses that closely matched the titers induced by intramuscular controls at the lower dose (6 μg). This may indicate that the delivery system developed in this study effectively adhered to nasal epithelial cells and successfully traversed the epithelial barrier to penetrate deeper tissues. This indicates that MHCS‐LNPs are likely to be captured by the APCs of the mucosal immune system, which then transport the administered antigens to immune initiation sites such as Peyer's patches, promoting efficient immune response induction. Even though at a 6‐μg dose, intramuscular administration resulted in a higher immune response than intranasal administration, in overall evaluation intranasal vaccines compensate for the shortcomings of intramuscular vaccines and can induce mucosal immune responses, improving the prevention of respiratory virus infection. Additionally, the data showed that the high dose (24 μg) did not exhibit any noticeable superiority compared to the median dose, and in most cases, the median dose resulted in stronger immunity. This may be due to the phenomenon known as the antigenic threshold, which refers to the point at which the immune response reaches saturation and further increases in antigen concentration do not result in a corresponding increase in antibody production. It is clear that investigating this phenomenon requires more evaluation. The application of a low dose represents a crucial advancement in minimizing potential off‐target effects and immunological side effects linked to mRNA doses.

These encouraging data from sera suggest that equivalent or improved results could be expected in mucosal immune response, considering the crucial function of IgA in mucosal immunity and its ability to neutralize influenza particles in the upper respiratory tract by preventing viral attachment to mucosal surfaces, as well as the requirement for antigen‐specific neutralizing antibodies in the lower respiratory tract to offer full protection against pathogens not inhibited in the upper respiratory tract. This study explored the capacity of the mRNA‐MHCS‐LNP vaccine to elicit IgA responses in the lower respiratory tract. As expected, the findings demonstrated that the mRNA‐MHCS‐LNP vaccine significantly boosted HA‐specific IgA levels in BAL fluids when compared to the control groups. This suggests that the vaccine candidate has the potential to stimulate mucosal immunity, serving as a primary defense mechanism and potentially helping to control the spread of influenza virus infection. Interestingly, intramuscular immunization did not lead to notable IgA responses in any of the mucosal samples tested, indicating that the intramuscular route is less effective at inducing mucosal immunity compared to intranasal administration. This is a key distinction, as mucosal immunity is crucial for protection at the site of pathogen entry, particularly for respiratory viruses like influenza. While intramuscular vaccines are effective in generating systemic immunity, they do not typically stimulate IgA responses in mucosal tissues, underscoring the advantages of intranasal vaccination for diseases that primarily affect mucosal surfaces.

Significantly, this research employed higher dosages for intranasal administration than for intramuscular delivery, aligning with recent findings indicating the necessity of increased dosages to effectively deliver an ample amount of product to target cells, including epithelial cells in the upper respiratory tract. This adjustment is likely due to the physiological barriers present in the mucosa [61, 62].

Evaluating intranasal vaccination as a booster regimen after primary parenteral vaccination schedules is also essential, as an intranasal booster may enhance mucosal immunity postprimary vaccination, offering early and long‐lasting protection against infections. Based on recent research by Mao et al. [63], intranasal vaccination at varying intervals (from days to months) following the initial intramuscular injection of the mRNA vaccine (known as the prime and spike approach) effectively induced robust protective mucosal immunity through the activation of CD8+ and CD4+ memory T cells, memory B cells, and IgA. Notably, this approach significantly reduced the viral load in both the upper and lower airways, thereby preventing disease and death from a lethal SARS‐CoV‐2 challenge.

This research will undoubtedly benefit from evaluations of influenza virus challenges in future studies. Bonney et al. reported that intranasal administration of a heat‐inactivated influenza virus successfully protected infant mice from a lethal challenge, while intramuscular administration did not provide the same level of protection [64]. Similarly, intranasal vaccination of mice with virus‐like particles (VLPs) from structural proteins of the pandemic 1918 Influenza A (H1N1) leads to protection against a lethal challenge with both the 1918 virus and the H5N1 virus; in contrast, mice that received intramuscular immunizations of 1918 VLPs were only protected against a challenge from a homologous virus [65]. These data highlight the potential for intranasal vaccines to offer superior protection against influenza infection compared to traditional intramuscular vaccines.

## 5 Conclusion

In conclusion, we demonstrated that intranasally administered mRNA‐MHCS‐LNPs as a two‐dose regimen are immunogenic and can elicit HA‐neutralizing antibodies in mice sera and BALF. Additionally, we have shown that chitosan nanoparticles can be tailored and formulated for improved respiratory delivery, which increases immunogenicity.

## Author Contributions

**Sara Yahyaei:** methodology, project administration, writing – review and editing, writing – original draft, formal analysis, investigation, software, funding acquisition. **Asghar Abdoli:** conceptualization, supervision, data curation, visualization, resources. **Abbas Jamali:** conceptualization, supervision. **Ali Teimoori:** conceptualization, supervision, software. **Ehsan Arefian:** conceptualization, supervision. **Zohre Eftekhari:** methodology, supervision, validation. **Parisa Jamur:** formal analysis, investigation.

## Conflicts of Interest

The authors declare no conflicts of interest.

## Supporting information


**Data S1** Supporting information.

## Data Availability

The data that support the findings of this study are available from the corresponding author upon reasonable request.

## References

[1] F. Krammer , “The Human Antibody Response to Influenza A Virus Infection and Vaccination,” Nature Reviews. Immunology 19, no. 6 (2019): 383–397. 10.1038/s41577-019-0143-6 30837674

[2] P. Palese and M. Shaw , “Fields Virology,” in Orthomyxoviridae: The Viruses and Their Replication, 5th ed. (Lippincott Williams & Wilkins, Wolters Kluwer Business, 2007): 1647–1689.

[3] I. Kosik and J. W. Yewdell , “Influenza Hemagglutinin and Neuraminidase: Yin^−^Yang Proteins Coevolving to Thwart Immunity,” Viruses 11, no. 4 (2019): 346. 31014029 10.3390/v11040346 PMC6520700

[4] V. N. Petrova and C. A. Russell , “The Evolution of Seasonal Influenza Viruses,” Nature Reviews. Microbiology 16, no. 1 (2018): 47–60. 29081496 10.1038/nrmicro.2017.118

[5] C. M. Trombetta , O. Kistner , E. Montomoli , S. Viviani , and S. Marchi , “Influenza Viruses and Vaccines: The Role of Vaccine Effectiveness Studies for Evaluation of the Benefits of Influenza Vaccines,” Vaccines (Basel). 10, no. 5 (2022): 714. 35632470 10.3390/vaccines10050714 PMC9143275

[6] K. S. Corbett , D. K. Edwards , S. R. Leist , et al., “SARS‐CoV‐2 mRNA Vaccine Design Enabled by Prototype Pathogen Preparedness,” Nature 586, no. 7830 (2020): 567–571. 32756549 10.1038/s41586-020-2622-0 PMC7581537

[7] K. Ali , G. Berman , H. Zhou , et al., “Evaluation of mRNA‐1273 SARS‐CoV‐2 Vaccine in Adolescents,” New England Journal of Medicine 385, no. 24 (2021): 2241–2251. 34379915 10.1056/NEJMoa2109522 PMC8385554

[8] A. B. Vogel , I. Kanevsky , Y. Che , et al., A Prefusion SARS‐CoV‐2 Spike RNA Vaccine is Highly Immunogenic and Prevents Lung Infection in Non‐Human Primates. bioRxiv. 2020:2020.09.08.280818.

[9] U. Sahin , A. Muik , I. Vogler , et al., “BNT162b2 Vaccine Induces Neutralizing Antibodies and Poly‐Specific T Cells in Humans,” Nature 595, no. 7868 (2021): 572–577. 34044428 10.1038/s41586-021-03653-6

[10] R. W. Frenck, Jr. , N. P. Klein , N. Kitchin , et al., “Safety, Immunogenicity, and Efficacy of the BNT162b2 Covid‐19 Vaccine in Adolescents,” New England Journal of Medicine 385, no. 3 (2021): 239–250. 34043894 10.1056/NEJMoa2107456 PMC8174030

[11] G. Guerrera , M. Picozza , S. D'Orso , et al., “BNT162b2 Vaccination Induces Durable SARS‐CoV‐2‐Specific T Cells With a Stem Cell Memory Phenotype,” Science Immunology 6, no. 66 (2021): eabl5344. 34726470 10.1126/sciimmunol.abl5344

[12] C. Iavarone , D. T. O'Hagan , D. Yu , N. F. Delahaye , and J. B. Ulmer , “Mechanism of Action of mRNA‐Based Vaccines,” Expert Review of Vaccines 16, no. 9 (2017): 871–881. 28701102 10.1080/14760584.2017.1355245

[13] O. Andries , S. Mc Cafferty , S. C. De Smedt , R. Weiss , N. N. Sanders , and T. Kitada , “N(1)‐Methylpseudouridine‐Incorporated mRNA Outperforms Pseudouridine‐Incorporated mRNA by Providing Enhanced Protein Expression and Reduced Immunogenicity in Mammalian Cell Lines and Mice,” Journal of Controlled Release 217 (2015): 337–344. 26342664 10.1016/j.jconrel.2015.08.051

[14] Y. V. Svitkin , Y. M. Cheng , T. Chakraborty , V. Presnyak , M. John , and N. Sonenberg , “N1‐Methyl‐Pseudouridine in mRNA Enhances Translation Through eIF2α‐Dependent and Independent Mechanisms by Increasing Ribosome Density,” Nucleic Acids Research 45, no. 10 (2017): 6023–6036. 28334758 10.1093/nar/gkx135 PMC5449617

[15] G. Zhang , T. Tang , Y. Chen , X. Huang , and T. Liang , “mRNA Vaccines in Disease Prevention and Treatment,” Signal Transduction and Targeted Therapy 8, no. 1 (2023): 365. 37726283 10.1038/s41392-023-01579-1 PMC10509165

[16] C. L. Gebhart and A. V. Kabanov , “Evaluation of Polyplexes as Gene Transfer Agents,” Journal of Controlled Release 73, no. 2 (2001): 401–416. 11516515 10.1016/s0168-3659(01)00357-1

[17] H. N. Abdelhamid , “Chitosan‐Based Nanocarriers for Gene Delivery,” Nanoengineering of Biomaterials 1 (2022): 91–105.

[18] D. W. Pack , D. Putnam , and R. Langer , “Design of Imidazole‐Containing Endosomolytic Biopolymers for Gene Delivery,” Biotechnology and Bioengineering 67, no. 2 (2000): 217–223. 10592519

[19] B. Layek and J. Singh , “Amino Acid Grafted Chitosan for High Performance Gene Delivery: Comparison of Amino Acid Hydrophobicity on Vector and Polyplex Characteristics,” Biomacromolecules 14, no. 2 (2013): 485–494. 23301560 10.1021/bm301720g

[20] C. Moreira , H. Oliveira , L. R. Pires , S. Simões , M. A. Barbosa , and A. P. Pêgo , “Improving Chitosan‐Mediated Gene Transfer by the Introduction of Intracellular Buffering Moieties Into the Chitosan Backbone,” Acta Biomaterialia 5, no. 8 (2009): 2995–3006. 19427930 10.1016/j.actbio.2009.04.021

[21] K.‐L. Chang , Y. Higuchi , S. Kawakami , F. Yamashita , and M. Hashida , “Efficient Gene Transfection by Histidine‐Modified Chitosan Through Enhancement of Endosomal Escape,” Bioconjugate Chemistry 21, no. 6 (2010): 1087–1095. 20499901 10.1021/bc1000609

[22] S. Renu , N. Feliciano‐Ruiz , V. Patil , et al., “Immunity and Protective Efficacy of Mannose Conjugated Chitosan‐Based Influenza Nanovaccine in Maternal Antibody Positive Pigs,” Frontiers in Immunology 12 (2021): 10. 10.3389/fimmu.2021.584299 PMC7969509 33746943

[23] I. A. Hajam , A. Senevirathne , C. Hewawaduge , J. Kim , and J. H. Lee , “Intranasally Administered Protein Coated Chitosan Nanoparticles Encapsulating Influenza H9N2 HA2 and M2e mRNA Molecules Elicit Protective Immunity Against Avian Influenza Viruses in Chickens,” Veterinary Research 51, no. 1 (2020): 37. 32143695 10.1186/s13567-020-00762-4 PMC7060564

[24] K. Tabynov , M. Solomadin , N. Turebekov , et al., “An Intranasal Vaccine Comprising SARS‐CoV‐2 Spike Receptor‐Binding Domain Protein Entrapped in Mannose‐Conjugated Chitosan Nanoparticle Provides Protection in Hamsters,” Scientific Reports 13, no. 1 (2023): 12115. 37495639 10.1038/s41598-023-39402-0 PMC10372096

[25] Y. Ma , Y. Zhao , R. Chen , et al., “Mucosal Immunity of Mannose‐Modified Chitosan Microspheres Loaded With the Nontyepable Haemophilus Influenzae Outer Membrane Protein P6 in BALB/c Mice,” PLoS ONE 17, no. 6 (2022): e0269153. 35687548 10.1371/journal.pone.0269153 PMC9187061

[26] B. Ma , S. Zhang , H. Jiang , B. Zhao , and H. Lv , “Lipoplex Morphologies and Their Influences on Transfection Efficiency in Gene Delivery,” Journal of Controlled Release 123, no. 3 (2007): 184–194. 17913276 10.1016/j.jconrel.2007.08.022

[27] G. Baldeon Vaca , M. Meyer , A. Cadete , et al., “Intranasal mRNA‐LNP Vaccination Protects Hamsters From SARS‐CoV‐2 Infection,” Science Advances 9, no. 38 (2023): eadh1655. 37738334 10.1126/sciadv.adh1655 PMC10516494

[28] O. P. Choudhary , M. T. A. Priyanka , and I. Singh , “Intranasal COVID‐19 Vaccines: Is It a Boon or Bane?,” International Journal of Surgery 94 (2021): 106119. 34536600 10.1016/j.ijsu.2021.106119 PMC8443315

[29] D. George , P. U. Maheswari , and K. M. M. S. Begum , “Chitosan‐Cellulose Hydrogel Conjugated With L‐Histidine and Zinc Oxide Nanoparticles for Sustained Drug Delivery: Kinetics and In‐Vitro Biological Studies,” Carbohydrate Polymers 236 (2020): 116101. 32172900 10.1016/j.carbpol.2020.116101

[30] Y. Han , S. Renu , V. Patil , et al., “Mannose‐Modified Chitosan‐Nanoparticle‐Based Salmonella Subunit OralVaccine‐Induced Immune Response and Efficacy in a Challenge Trial in Broilers,” Vaccine 8, no. 2 (2020): 299. 10.3390/vaccines8020299 PMC7349978 32545295

[31] F. Szoka, Jr. and D. Papahadjopoulos , “Procedure for Preparation of Liposomes With Large Internal Aqueous Space and High Capture by Reverse‐Phase Evaporation,” Proceedings of the National Academy of Sciences of the United States of America 75, no. 9 (1978): 4194–4198. 279908 10.1073/pnas.75.9.4194 PMC336078

[32] O. Mertins , M. Sebben , A. R. Pohlmann , and N. P. da Silveira , “Production of Soybean Phosphatidylcholine–Chitosan Nanovesicles by Reverse Phase Evaporation: A Step by Step Study,” Chemistry and Physics of Lipids 138, no. 1 (2005): 29–37. 16144696 10.1016/j.chemphyslip.2005.07.004

[33] Ö. Tezgel , A. Szarpak‐Jankowska , A. Arnould , R. Auzély‐Velty , and I. Texier , “Chitosan‐Lipid Nanoparticles (CS‐LNPs): Application to siRNA Delivery,” Journal of Colloid and Interface Science 510 (2018): 45–56. 28934610 10.1016/j.jcis.2017.09.045

[34] B. B. M. Garcia , O. Mertins , E. R. D. Silva , P. D. Mathews , and S. W. Han , “Arginine‐Modified Chitosan Complexed With Liposome Systems for Plasmid DNA Delivery,” Colloids and Surfaces. B, Biointerfaces 193 (2020): 111131. 32512370 10.1016/j.colsurfb.2020.111131

[35] R. K. Dhandapani , D. Gurusamy , J. L. Howell , and S. R. Palli , “Development of CS‐TPP‐dsRNA Nanoparticles to Enhance RNAi Efficiency in the Yellow Fever Mosquito, *Aedes aegypti* ,” Scientific Reports 9, no. 1 (2019): 8775. 31217512 10.1038/s41598-019-45019-z PMC6584730

[36] R. E. McKenzie , J. J. Minnell , M. Ganley , G. F. Painter , and S. L. Draper , “mRNA Synthesis and Encapsulation in Ionizable Lipid Nanoparticles,” Current Protocols 3, no. 9 (2023): e898. 37747354 10.1002/cpz1.898

[37] P. Zamani , M. Mashreghi , M. Rezazade Bazaz , et al., “Characterization of Stability, Safety and Immunogenicity of the mRNA Lipid Nanoparticle Vaccine Iribovax® Against COVID‐19 in Nonhuman Primates,” Journal of Controlled Release 360 (2023): 316–334. 37355212 10.1016/j.jconrel.2023.06.025

[38] L. Karpenko , A. Rudometov , S. Sharabrin , et al., “Delivery of mRNA Vaccine against SARS‐CoV‐2 Using a Polyglucin:Spermidine Conjugate,” Vaccine 9 (2021): 76. 10.3390/vaccines9020076 PMC7910849 33494530

[39] T. L. Bricker , T. L. Darling , A. O. Hassan , et al., “A Single Intranasal or Intramuscular Immunization With Chimpanzee Adenovirus‐Vectored SARS‐CoV‐2 Vaccine Protects Against Pneumonia in Hamsters,” Cell Reports 36, no. 3 (2021): 109400. 34245672 10.1016/j.celrep.2021.109400 PMC8238649

[40] A. M. Alnuqaydan , A. Almutary , G. R. Bhat , et al., “Evaluation of the Cytotoxic, Anti‐Inflammatory, and Immunomodulatory Effects of Withaferin A (WA) Against Lipopolysaccharide (LPS)‐Induced Inflammation in Immune Cells Derived From BALB/c Mice,” Pharmaceutics. 14, no. 6 (2022): 4. 10.3390/pharmaceutics14061256 PMC9229769 35745829

[41] N. Pardi , K. Parkhouse , E. Kirkpatrick , et al., “Nucleoside‐Modified mRNA Immunization Elicits Influenza Virus Hemagglutinin Stalk‐Specific Antibodies,” Nature Communications 9, no. 1 (2018): 3361. 10.1038/s41467-018-05482-0 PMC6105651 30135514

[42] A. Sabbaghi , M. Malek , S. Abdolahi , et al., “A Formulated Poly (I:C)/CCL21 as an Effective Mucosal Adjuvant for Gamma‐Irradiated Influenza Vaccine,” Virology Journal 18, no. 1 (2021): 201. 34627297 10.1186/s12985-021-01672-3 PMC8501930

[43] C. R. Norris , J. R. Byerly , K. C. Decile , et al., “Allergen‐Specific IgG and IgA in Serum and Bronchoalveolar Lavage Fluid in a Model of Experimental Feline Asthma,” Veterinary Immunology and Immunopathology 96, no. 3 (2003): 119–127. 14592725 10.1016/s0165-2427(03)00144-2

[44] T. Sato , A. Kitajima , S. Ohmoto , et al., “Determination of Human Immunoglobulin A and Secretory Immunoglobulin A in Bronchoalveolar Lavage Fluids by Solid Phase Enzyme Immunoassay,” Clinica Chimica Acta 220, no. 2 (1993): 145–156. 10.1016/0009-8981(93)90043-4 8111959

[45] X. Zhuang , Y. Qi , M. Wang , et al., “mRNA Vaccines Encoding the HA Protein of Influenza A H1N1 Virus Delivered by Cationic Lipid Nanoparticles Induce Protective Immune Responses in Mice,” Vaccines (Basel). 8, no. 1 (2020): 6. 10.3390/vaccines8010123 PMC7157730 32164372

[46] J. C. Pedersen , “Hemagglutination‐Inhibition Assay for Influenza Virus Subtype Identification and the Detection and Quantitation of Serum Antibodies to Influenza Virus,” Methods in Molecular Biology 1161 (2014): 11–25. 24899416 10.1007/978-1-4939-0758-8_2

[47] M. Zacour , B. J. Ward , A. Brewer , et al., “Standardization of Hemagglutination Inhibition Assay for Influenza Serology Allows for High Reproducibility Between Laboratories,” Clinical and Vaccine Immunology 23, no. 3 (2016): 236–242. 26818953 10.1128/CVI.00613-15 PMC4783428

[48] M. L. Killian , “Hemagglutination Assay for Influenza Virus,” Methods in Molecular Biology 1161 (2014): 3–9. 24899415 10.1007/978-1-4939-0758-8_1

[49] H. F. Ross , “Scattering and Particle Sizing Applications*,” in Encyclopedia of Spectroscopy and Spectrometry, Second ed., ed. J. C. Lindon (Academic Press, 1999): 2488–2494.

[50] X. Z. Shu and K. J. Zhu , “A Novel Approach to Prepare Tripolyphosphate/Chitosan Complex Beads for Controlled Release Drug Delivery,” International Journal of Pharmaceutics 201, no. 1 (2000): 51–58. 10867264 10.1016/s0378-5173(00)00403-8

[51] S. Prabha , G. Arya , R. Chandra , B. Ahmed , and S. Nimesh , “Effect of Size on Biological Properties of Nanoparticles Employed in Gene Delivery,” Artificial Cells, Nanomedicine, and Biotechnology 44, no. 1 (2016): 83–91. 24866724 10.3109/21691401.2014.913054

[52] A. Nair , M. A. Morsy , and S. Jacob , “Dose Translation Between Laboratory Animals and Human in Preclinical and Clinical Phases of Drug Development,” Drug Development Research 79, no. 8 (2018): 373–382. 30343496 10.1002/ddr.21461

[53] J. Tang , C. Zeng , T. M. Cox , et al., “Respiratory Mucosal Immunity Against SARS‐CoV‐2 After mRNA Vaccination,” Science Immunology 7, no. 76 (2022): eadd4853. 35857583 10.1126/sciimmunol.add4853 PMC9348751

[54] A. Billich , “Technology Evaluation: FluMist, University of Michigan,” Current Opinion in Molecular Therapeutics 2, no. 3 (2000): 340–344. 11249630

[55] D. S. Rajão and D. R. Pérez , “Universal Vaccines and Vaccine Platforms to Protect Against Influenza Viruses in Humans and Agriculture,” Frontiers in Microbiology 9 (2018): 123. 29467737 10.3389/fmicb.2018.00123 PMC5808216

[56] H. Yusuf and V. Kett , “Current Prospects and Future Challenges for Nasal Vaccine Delivery,” Human Vaccines & Immunotherapeutics 13, no. 1 (2017): 34–45. 27936348 10.1080/21645515.2016.1239668 PMC5287317

[57] C. Foged , B. Brodin , S. Frokjaer , and A. Sundblad , “Particle Size and Surface Charge Affect Particle Uptake by Human Dendritic Cells in an In Vitro Model,” International Journal of Pharmaceutics 298, no. 2 (2005): 315–322. 15961266 10.1016/j.ijpharm.2005.03.035

[58] S. Kumar , A. C. Anselmo , A. Banerjee , M. Zakrewsky , and S. Mitragotri , “Shape and Size‐Dependent Immune Response to Antigen‐Carrying Nanoparticles,” Journal of Controlled Release 220, no. Pt A (2015): 141–148. 26437263 10.1016/j.jconrel.2015.09.069

[59] S. D. Xiang , A. Scholzen , G. Minigo , et al., “Pathogen Recognition and Development of Particulate Vaccines: Does Size Matter?,” Methods 40, no. 1 (2006): 1–9. 16997708 10.1016/j.ymeth.2006.05.016

[60] Y. Su , B. Sun , X. Gao , et al., “Intranasal Delivery of Targeted Nanoparticles Loaded With miR‐132 to Brain for the Treatment of Neurodegenerative Diseases,” Frontiers in Pharmacology 11 (2020): 1165. 32848773 10.3389/fphar.2020.01165 PMC7424054

[61] M. Madhavan , A. J. Ritchie , J. Aboagye , et al., “Tolerability and Immunogenicity of an Intranasally‐Administered Adenovirus‐Vectored COVID‐19 Vaccine: An Open‐Label Partially‐Randomised Ascending Dose Phase I Trial,” eBioMedicine 85 (2022): 104298. 36229342 10.1016/j.ebiom.2022.104298 PMC9550199

[62] N. van Doremalen , J. N. Purushotham , J. E. Schulz , et al., “Intranasal ChAdOx1 nCoV‐19/AZD1222 Vaccination Reduces Viral Shedding After SARS‐CoV‐2 D614G Challenge in Preclinical Models,” Science Translational Medicine 13, no. 607 (2021): eabh0755. 34315826 10.1126/scitranslmed.abh0755 PMC9267380

[63] T. Mao , B. Israelow , M. A. Peña‐Hernández , et al., “Unadjuvanted Intranasal Spike Vaccine Elicits Protective Mucosal Immunity Against Sarbecoviruses,” Science 378, no. 6622 (2022): eabo2523. 36302057 10.1126/science.abo2523 PMC9798903

[64] E. A. Bonney , K. Krebs , J. Kim , et al., “Protective Intranasal Immunization Against Influenza Virus in Infant Mice Is Dependent on IL‐6,” Frontiers in Immunology 11 (2020): 568978. 33193346 10.3389/fimmu.2020.568978 PMC7656064

[65] L. A. Perrone , A. Ahmad , V. Veguilla , et al., “Intranasal Vaccination With 1918 Influenza Virus‐Like Particles Protects Mice and Ferrets From Lethal 1918 and H5N1 Influenza Virus Challenge,” Journal of Virology 83, no. 11 (2009): 5726–5734. 19321609 10.1128/JVI.00207-09 PMC2681940

