# Supplementary material for: Targeting Respiratory Viruses: The Efficacy of Intranasal mRNA Vaccination in Generating Protective Mucosal and Systemic Immunity Against Influenza A (H1N1)
Source: Influenza Other Respir Viruses. 2025 Mar 24;19(3):e70093. doi: 10.1111/irv.70093 (PMC11932742; doi:10.1111/irv.70093)
Supplement: Supplementary file 1 — Data S1 Supporting information. [file IRV-19-e70093-s001.docx]

**Supplementary File**

**Supplementary Method:**

- 1. **Bioinformatic studies and template plasmid construction**

The full-length gene and protein sequence of the influenza A (H1N1) virus hemagglutinin glycoprotein were obtained from accession numbers FJ966082 and ACP41105. The prefusion-stabilized hemagglutinin protein was designed with two proline residue substitution mutations at V399P and S415P. The human codon-optimized coding region is downstream of the T7 bacteriophage promoter and flanked by the 5′-UTR, 3′-UTR, and 110 adenine nucleotides, interrupted by a 10-nucleotide linker, such as the poly(A) tail. The whole construct was inserted into the PUC57 plasmid and transformed into the E. coli strain DH5α for plasmid amplification. Also, to generate a reporter mRNA, the optimized coding sequence of Green fluorescent protein (GFP) for expression in mammalian cells was employed in the construct.

- 1. **In vitro mRNA transcription**

mRNA was transcribed using Hi-T7® RNA Polymerase (NEB, cat NO. M0658S) and N1-methyl pseudouridine-5’-triphosphate (m1ΨTP), the modified nucleoside (Jena Bioscience, cat NO. NU-890S) and capped with the Vaccinia capping system (NEB, cat NO. M2080S). Subsequently, the produced mRNA was purified with a Monarch® RNA Cleanup kit (NEB, cat NO. T2037L). The size and integrity of the transcribed mRNA were controlled by native agarose gel electrophoresis, and the concentration was calculated by measuring the absorbance at 260 nm using a Nanodrop 2000 spectrophotometer.

- 1. **Conjugation of chitosan with Histidine**

Histidine-conjugated chitosan polymers were produced by interacting with a reactive carboxylic acid group in histidine and the available primary amino groups in chitosan utilizing EDC (Sigma–Aldrich) and NHS agents (Sigma–Aldrich). Briefly, 2 g of chitosan (Mw= 100 KDa) was dissolved thoroughly overnight in a 1% acetic acid solution to achieve a 1 mg/ml concentration and pH of 3.5. Then, 0.6 g of L-histidine (0.3 mol/mol D-glucosamine of chitosan) was slowly added to the chitosan solution. In the next step, 0.5 g of EDC crosslinker (0.8 mol/mol of amino acid) was added while continuously stirring the mixture. The deprotonating base trimethylamine was present during the addition of the crosslinker. Trimethylamine helps to activate the EDC crosslinker by deprotonating the carboxylic acid group, which then reacts with the primary amine group of the molecule of interest. The reaction mixture was neutralized using 3 N sodium hydroxide solution, and the resulting precipitate was repeatedly washed with distilled water until neutral conditions were reached. Finally, the HCS was washed with acetone, lyophilized, and stored under the mentioned conditions [29].

- 1. **Conjugation of D-mannose to HCSs**

Mannose conjugation to HCSs was achieved through a reductive amination process in the presence of sodium triacetoxyborohydride. First, with stirring, 500 mg of C-Hi was dissolved in 1% aqueous acetic acid (pH 5.5). To prepare the D-mannose (Sigma–Aldrich) and sodium triacetoxyborohydride (Alfa Aesar, cat no. B22060) mixture, 100 mg of each mixture was dissolved in 0.5 mL of borate buffer. The resulting mixture was gradually added to HCSs under magnetic stirring for 72 hours at 56 °C. The mannose-conjugated HCS (MHCS) was dialyzed against double distilled water and freeze-dried[30]. FT-IR spectroscopy was used to analyze the chemical structure of HCSs and MHCSs over the range of 4000–400 cm−1, and subsequently, the degree of substitution (DS) was calculated[20].

- 1. **Synthesis of chitosan-lipid nanoparticles (MHCS-LNPs)**

Chitosan-lipid nanoparticles were prepared using the reversed-phase evaporation method. First, MHCS was dissolved in a 1% acetic acid solution to obtain a 0.5% stock solution, which was subsequently diluted in 200 mM sodium acetate buffer (pH=4). The polyplex between MHCS and mRNA was formed by mixing capped mRNA (1 mg in 5 ml of RNase-free water) in 5 ml of MHCS solution at different N:P ratios ranging from 4:1 to 12:1 under gentle stirring for 30 minutes. The N/P ratio is the proportion of nitrogen groups on the nanoparticles, which carry a positive charge, to the phosphate groups on the mRNA molecule, which carry a negative charge. Subsequently, the polyplex particles were concentrated by centrifugation at 8000 × g for 10 min and resuspended in 2 ml of 0.2 M sodium acetate buffer (pH= 4.5). Next, stock solutions of lipids, phospholipid 90G (PC-90G) (Lipoid GmbH), and cholesterol (CHO) (Alfa Aesar Cat no. A11470) were prepared in chloroform. The organic phase contained 85 µmol of PC-90G and 65 µmol of CHO in 10 ml of diethyl ether. Subsequently, 0.25 ml of 10 mM Tris-HCl buffer (pH 7.5) containing 1 mM EDTA was added. The blend was subjected to sonication in a bath-type sonicator for 45 seconds. The organic solvents were then evaporated at approximately 500 mmHg for 10 minutes to produce a gel. Next, a polyplex solution containing 20% glycerol was added to the gel and vortexed for 30 s. The organic solvents were once more evaporated at approximately 700 mmHg at 26 °C for 30 minutes [31, 32]. This process removed the remaining organic solvents, resulting in the formation of a homogeneous suspension of liposomes. Finally, the nanoparticles were dialyzed against 100 mM acetate buffer (pH=5.5).

- 1. **Nanoparticle characterization**

The hydrodynamic diameter, polydispersity (PDI), and zeta potential of the MHCS-LNPs were analyzed using dynamic light scattering (DLS) at an angle of 173° with a Zeta Sizer Nano instrument (Malvern Instruments, UK). The measurements were performed in triplicate at a ratio of 1:100 in 10 mM NaCl at 25 ℃. [33, 34].

- 1. **Encapsulation efficiency**

The encapsulation efficiency was determined by fluorescence spectroscopy and conversion of the fluorescence intensity of the sample and standard RNAs to concentrations. Eight different concentrations of rRNA standard solutions (1000, 500, 250, 125, 62.5, 31.25, 15.625, and 0 ng/mL) were prepared in Tris-EDTA buffer. The mRNA-MHCS-LNPs were similarly eluted in Tris-EDTA buffer to acquire a sample with an approximate mRNA concentration of 250 ng/ml. Equivalent samples were also prepared in Tris-EDTA buffer with the addition of Triton-X100 surfactant (0.5%). Then, 100 µL of each sample, including standard solution and mRNA-MHCS-LNP samples, was dispensed into the microwells of a 96-well plate. Subsequently, 100 µL of 50 µM SYBR Green II was added. After a 5-minute incubation, the fluorescence intensity was measured using a fluorescence spectroscopy plate reader with the excitation and emission wavelengths set at 490 nm and 520 nm, respectively. A standard curve, created using standard mRNA solutions, was utilized to translate fluorescence intensity into mRNA concentration. The fluorescence obtained from mRNA-MHCS-LNP samples dispersed in Tris-EDTA buffer/Triton-X100 and Tris-EDTA buffer was assumed to represent total mRNAs and free (unencapsulated), respectively. The encapsulation efficiency (%EE) was calculated as in (1), where W(total) is the initiating concentration of mRNA and W(free.) is the encapsulated mRNA concentration: [35, 36]

%EE= $\frac{W\left( total mRNA \right)-W(free mRNA)}{W(total mRNA)}\times100$ (1)

- 1. **In vitro transfection efficiency**

To assess the transfection efficiency of the mRNA-MHCS-LNPs, three different cell lines, namely, HEK 293T cells, which are commonly used cell lines with a high propensity for transfection; A549 cells, which are representative of lung epithelial cells; and Raw264.7 cells, which are murine macrophage lines, were used. All cells were cultured in Dulbecco’s modified Eagle’s medium (DMEM) supplemented with 10% fetal bovine serum and incubated at 37 °C with 5% CO2. A total of 50,000 cells/well were seeded in 24-well culture plates in DMEM supplemented with 10% FBS and GFP coding-mRNA-MHCS-LNPs encapsulated with 1 µg of mRNA transfected in each well. As positive and negative controls, 1 µg of mRNA was transfected with Lipofectamine 3000 transfection reagent (Invitrogen-Catalog number L3000001) according to the manufacturer’s instructions and with empty MHCS-LNPs, respectively. After 48 h, the cells were observed via fluorescence microscopy and analyzed via flow cytometry, and the results were analyzed via FlowJo V10 software.

- 1. **Stability and storability of the mRNA-MHCS-LNPs**

To assess the stability and shelf life of MHCS-LNP-encapsulated mRNA, parameters including %EE, size, and zeta potential were measured following storage periods of 30 days in a refrigerator (2-8 °C) or a freezer (-20 °C). Parameters were investigated at 7, 14, and 30 days[37].

- 1. **In vitro cytotoxicity of the mRNA-MHCS-LNPs**

The XTT assay on the HEK-293T and A549 cell lines assessed the cytotoxicity of the prepared nanoparticles. Cells were seeded in a 96-well plate, and four concentrations of mRNA-MHCS-LNPs containing 0.25, 0.5, 1, or 2 µg of mRNA were transfected in triplicate. At 24 and 72 h post-transfection, the percentage of viable cells relative to that of PBS-transfected control cells (100% cell viability) was calculated using GraphPad Prism software. Statistical significance was calculated by one-way ANOVA[38].

- 1. **Animal studies**

The animal experiments and procedures were performed in accordance with the applicable guidelines and regulations, as approved by the ethical committee and in line with the ARRIVE guidelines. The animals used in the study were obtained from the Pasteur Institute of Iran and were maintained under standard laboratory conditions. During the postimmunization period, the animals were monitored for clinical signs, mortality, and changes in body weight. Six- to eight-week-old female BALB/c mice were randomly divided into six groups (n = 8): on day zero, the mRNA-MHCS-LNPs containing 6 µg of mRNA (low-dose), 12 µg of mRNA (medium-dose) or 24 µg of mRNA (high-dose) were intranasally administered. For the intranasal groups, the mice were anesthetized with ketamine (100 mg/kg) and xylazine (10 mg/kg), and the total volume of nanoparticle solution was 30 µL (15 µL in each nostril). One group was intramuscularly administered mRNA-MHCS-LNPs containing 6 µg of mRNA into the hind leg. Two other groups were employed as the control groups to receive either **50 µl** PBS or intramuscular administration of the commercial influenza vaccine (**VAXIGRIP Tetra**) (according to the dose conversion and weight between mice and humans). Fourteen days later, the mice were boosted with equal doses of the formulations. First, blood samples were collected at 4 weeks postimmunization for further analysis, and BALF samples were obtained from each mouse after death. The trachea was exposed, and a feeding needle was introduced into it and secured. A syringe was connected to the needle to perform three cycles of instilling and withdrawing 500 µl of a solution composed of PBS with 1% BSA and 1 mM PMSF. The collected BALF samples were chilled on ice and spun at 3000 ×g and 4 °C for 10 minutes. The resulting supernatants were either used for immediate testing or frozen at −20 °C for later analysis. Also, spleens were collected from each mouse under sterile conditions and were minced in the DMEM medium. The cell mixture, which included red blood cells (RBCs), was treated with a 0.8% ammonium chloride solution to induce lysis. Following this, the cell-ammonium chloride mixture was spun down in a centrifuge at 380× g for 10 minutes. The resulting cell pellet was re-suspended in DMEM medium that had been enriched with 10% fetal bovine serum (FBS) and 1% penicillin-streptomycin. The study was approved by the Research Ethics Committees (Approval ID: IR.PII.REC.1400.067, Approval Date: 2021-12-11).

- 1. **Evaluation of humoral immune responses**

The influenza A (H1N1) hemagglutinin-specific IgG antibody titers were evaluated via indirect enzyme-linked immunosorbent assay (ELISA). The collected blood samples were allowed to clot at room temperature and centrifuged for 10 min at 5000 × g, after which the serum samples were collected. First, Influenza H1N1/ A/California/04/09 Hemagglutinin glycoprotein was coated on a 96-well microplate. For this purpose, hemagglutinin protein was diluted in the bicarbonate/carbonate coating buffer (100mM, pH 9.6) at the final concentration of 5 ug/ml. 100µl of diluted protein was added to each well and incubated overnight at 4℃. Subsequently, wells were washed 3 times with PBS, and the remaining protein binding sites were blocked by the addition of 150µl of the ELISA blocking buffer( 1% Bovine serum albumin (BSA)). After 1h of incubation at room temperature, serial dilutions of mice sera in PBS were added and incubated for 2 h, followed by the addition of 1:100 diluted HRP-conjugated anti-mouse IgG. After 1 h of incubation, the substrate, tetramethylbenzidine (TMB), and stop solutions were added, and the absorbance of each well was measured using an ELISA reader at 450 nm. All sera were analyzed in duplicate[41].

- 1. **Evaluation of cell-mediated immune responses**

The concentrations of IL-5 (Mouse IL-5 CytoSet. Invitrogen, Germany, Cat.NO.BMS610TEN) and IFN-γ (DuoSet Mouse IFN-gamma, R&D Systems, Cat.NO DY485) in the collected splenocytes were analyzed using commercial mouse cytokine ELISA kits according to the manufacturer’s protocols. During this process, on day 28 after the first immunization, the mice were euthanized, and the spleens were collected aseptically. Splenocytes were seeded in a 96 Microwell plate at a density of 2 × 10^6^ cells per well. The cells were stimulated with the influenza hemagglutinin peptide pool (PepMix™ Influenza A (HA /California (H1N1)- (JPT Peptide Technologies)) at a final concentration of 5 μg/ml. The positive and negative controls used were a cell activation cocktail and RPMI 1640 medium, respectively. The supernatants were harvested 48 to 72 hours post-stimulation and then assessed for the presence of IFN-γ and IL-5 cytokines using a quantitative sandwich ELISA. The plates were measured at a wavelength of 450 nm, and the outcomes are displayed as optical densities. The concentration of cytokines in each sample was ascertained by comparing the OD values to a standard curve generated with known amounts of cytokines. Each test was performed in triplicate for every mouse. [42].

- 1. **Evaluation of mucosal IgA responses**

The influenza A (H1N1) hemagglutinin-specific mouse IgA indirect ELISA kit was adapted to identify antigen-specific IgA antibodies in the BALF samples. In summary, 100 µl of the BALF samples diluted 1:4 in the washing buffer ( PBS / Tween 20, 0.2%), were added to each well of a microtiter plate pre-coated with the 5µg/ml of Influenza H1N1/ A/California/04/09 Hemagglutinin glycoprotein, as described in 2.12 section, and the plate was left to incubate at 37 °C for 60 minutes. Subsequently, goat anti-mouse IgA-HRP (diluted 1:20000, from Abcam, USA) was added to the wells. Following another wash step, the plate was developed using TMB. The reaction was subsequently stopped by introducing a 2 M sulfuric acid solution. The absorbance was measured at 450 nm, with a reference wavelength of 630 nm, using a microplate reader[43, 44].

- 1. **Hemagglutination inhibition (HI) assay**

Hemagglutination inhibition (HI) assays were carried out according to standard procedures. **Initially, chicken blood was collected in an equal volume of Alsever's solution, and PBS was added. The mixture was then centrifuged at 800 × g for 10 minutes. The supernatant was discarded, and the erythrocytes were washed. This washing process was repeated for a total of three washes and a 0.5% suspension of packed chicken erythrocytes in PBS was prepared.** ~~to neutralize nonspecific inhibitors, serum samples were subjected to treatment with a receptor-destroying enzyme.~~ Formaldehyde inactivated influenza A/California/07/2009(H1N1) and A/Puerto Rico/8/1934 H1N1 strains gifted from the Influenza and Other Respiratory Viruses Department of Pasteur Institute of Iran. First, 50 µL of PBS was added to wells 1 to 12 of each row in a U-bottom 96-well microtiter plate. Then, 50 µL of influenza virus was added to the first well, and a serial 2-fold dilution was performed from wells 1 to 12. Next, 50 µL of a 0.5% erythrocyte suspension was added to each well, and the plate was incubated at room temperature for 20–30 minutes to allow the RBCs to settle. The HA titration endpoint is the last well where complete hemagglutination occurs, indicating the presence of 1 HA unit of the virus. Due to the 2-fold dilutions, the well two steps ahead of the HA titration endpoint contains 4 HA units of virus, which is used in the HI assay. Mice sera were diluted 1:10 in PBS and serial two-fold dilutions were prepared in wells 1 to 12. These dilutions were then incubated for 30 minutes at room temperature with 25 μL of virus containing 4 HA units. A 50 µL volume of 0.5% chicken RBCs was added, and the reaction was incubated for an additional 30 minutes at room temperature.~~In 96-well plates, twofold serial dilutions of sera were incubated with inactivated whole influenza A virus for 30 min. Subsequently, 0.5% (vol/vol) turkey red blood cells were added.~~ The final serum dilution at which complete HI was detected was determined visually, defined as the HI titer[45-48].
